# Supplementary material for: Genotyping-by-sequencing-based identification of Arabidopsis pattern recognition receptor RLP32 recognizing proteobacterial translation initiation factor IF1
Source: Nat Commun. 2022 Mar 11;13:1294. doi: 10.1038/s41467-022-28887-4 (PMC8917236; doi:10.1038/s41467-022-28887-4)
Supplement: Supplementary file 1 — Supplementary Information [file 41467_2022_28887_MOESM1_ESM.pdf]

Supplementary Information

Title : Genotyping-by-sequencing-based identification of *Arabidopsis* pattern recognition receptor RLP32 recognizing proteobacterial translation initiation factor IF1

Authors : Li Fan, Katja Fröhlich, Eric Melzer, Rory N. Pruitt, Isabell Albert, Lisha Zhang, Anna Joe, Chenlei Hua, Yanyue Song, Markus Albert, Sang-Tae Kim, Detlef Weigel, Cyril Zipfel, Eunyoung Chae, Andrea A. Gust, Thorsten Nürnberger

Supplementary figures

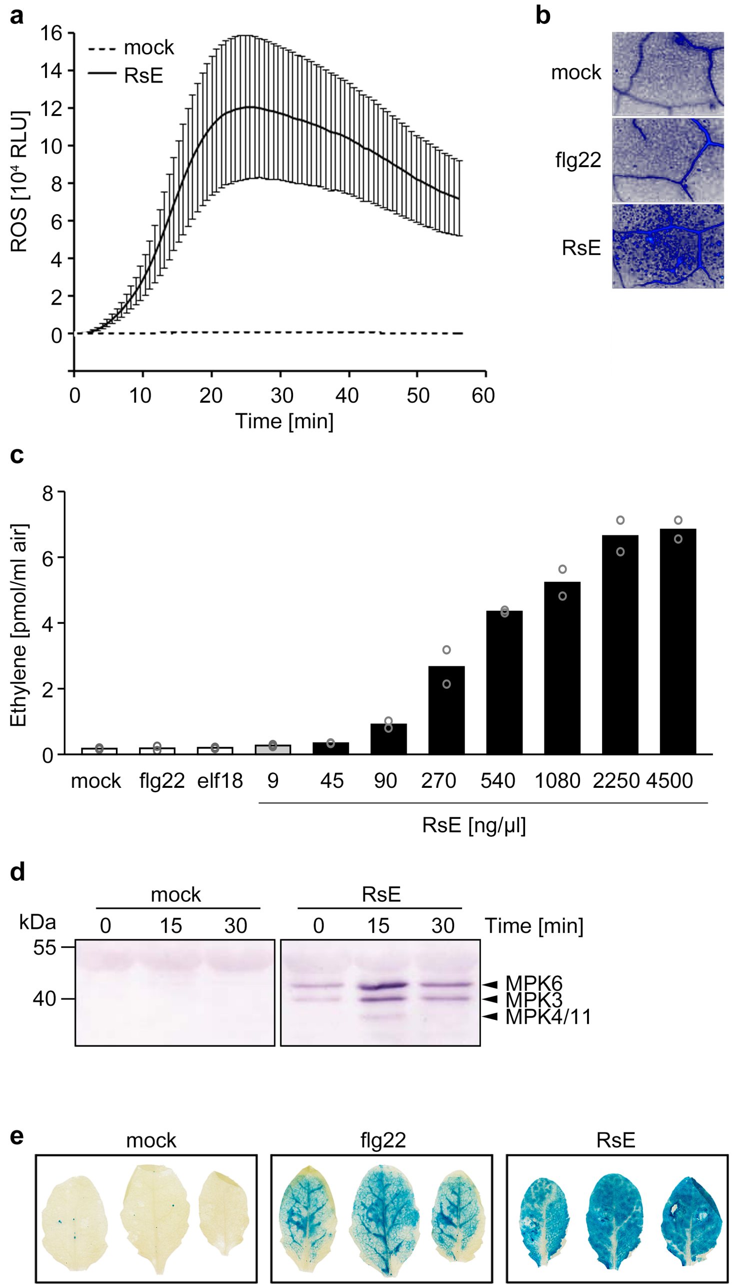

Supplementary Figure 1. **RsE induces plant immune responses in *Arabidopsis*. a** ROS accumulation in leaf pieces of *Arabidopsis fls2 efr* plants treated with water (mock), or RsE. Given are relative light units (RLU) ± SD (*n*=6 biologically independent samples for mock and RsE treatments). **b** Aniline blue stain of callose appositions 24 h after treatment of *fls2 efr* leaves with water (mock), flg22, or RsE. **c** Ethylene production in *Arabidopsis fls2 efr* plants treated with increasing RsE concentrations. Water treatment (mock) or treatment with flg22 or elf18 served as controls. Data points indicate two biologically independent samples. **d** *Arabidopsis fls2 efr* plants were treated for the times indicated with water (mock) or RsE. MAPK activation was detected by immunoblot using phospho-p44/p42 antibodies. **e** *pPR1::GUS* induction in leaves of three *pPR1::GUS* transgenic *Arabidopsis* lines infiltrated for 24 h with water (mock), flg22 or RsE. Experiments were performed twice (**b**-**d**) or three times (**a**, **e**) with similar results.

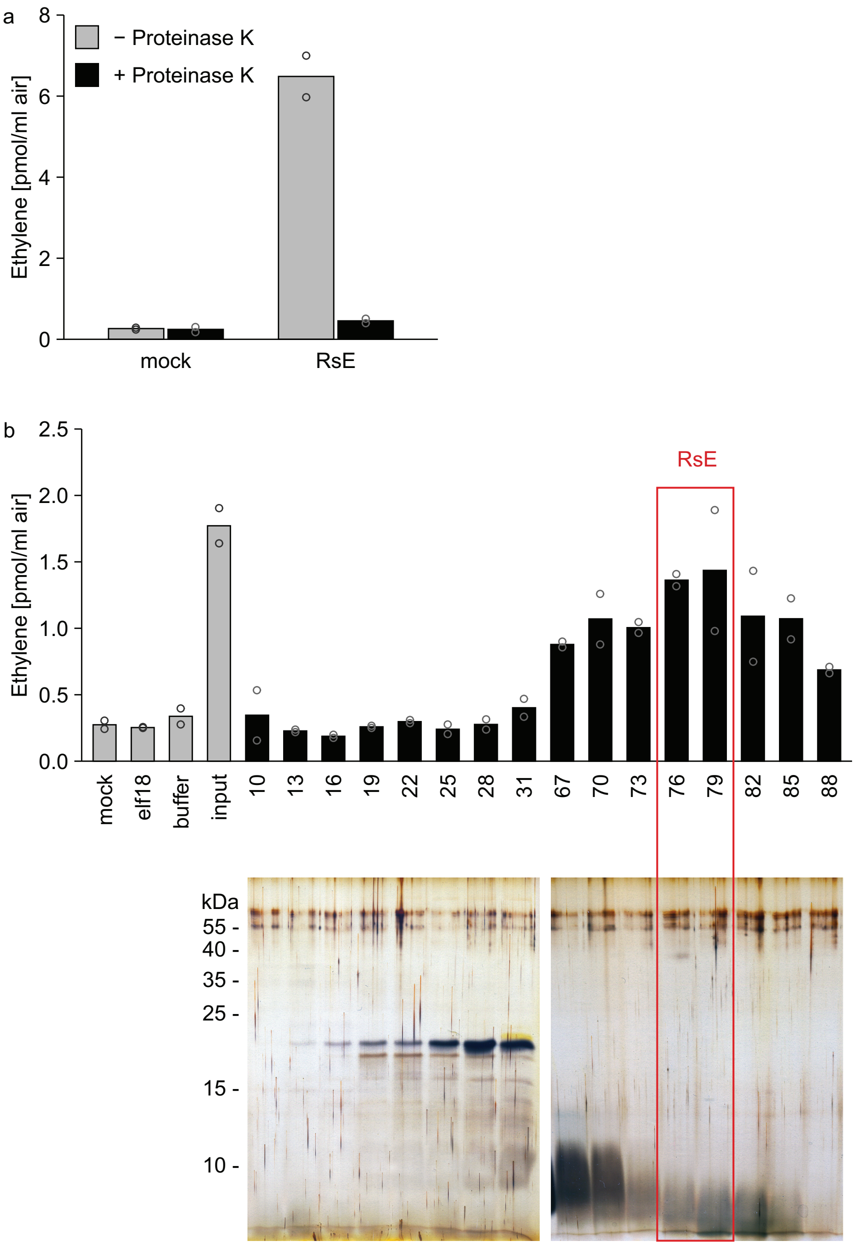

Supplementary Figure 2. **RsE elicitor activity is protease-sensitive and co-migrates with fractions containing low molecular weight proteins. a** Ethylene accumulation in *Arabidopsis fls2 efr* leaf pieces treated with water (mock) or RsE incubated for 2 h with 0,5 µg/µl Proteinase K (+) or left untreated (-). n=2 biologically independent samples for mock and RsE treatments. **b** Ethylene accumulation (upper panel) in *Arabidopsis fls2 efr* leaf pieces treated with water (mock), elf18, buffer, *R. solanacearum* cell extract (input) or with fractions obtained by gel filtration (number of fraction indicated). Data points indicate two replicates. Comparison with the elution profile of proteins with known size indicated that highest elicitor activity co-migrates with molecular masses <10 kDa (red box). Gel filtration fractions were analyzed by Tricine-SDS-PAGE followed by silver staining (lower panels). Experiments were performed at least twice with similar results.

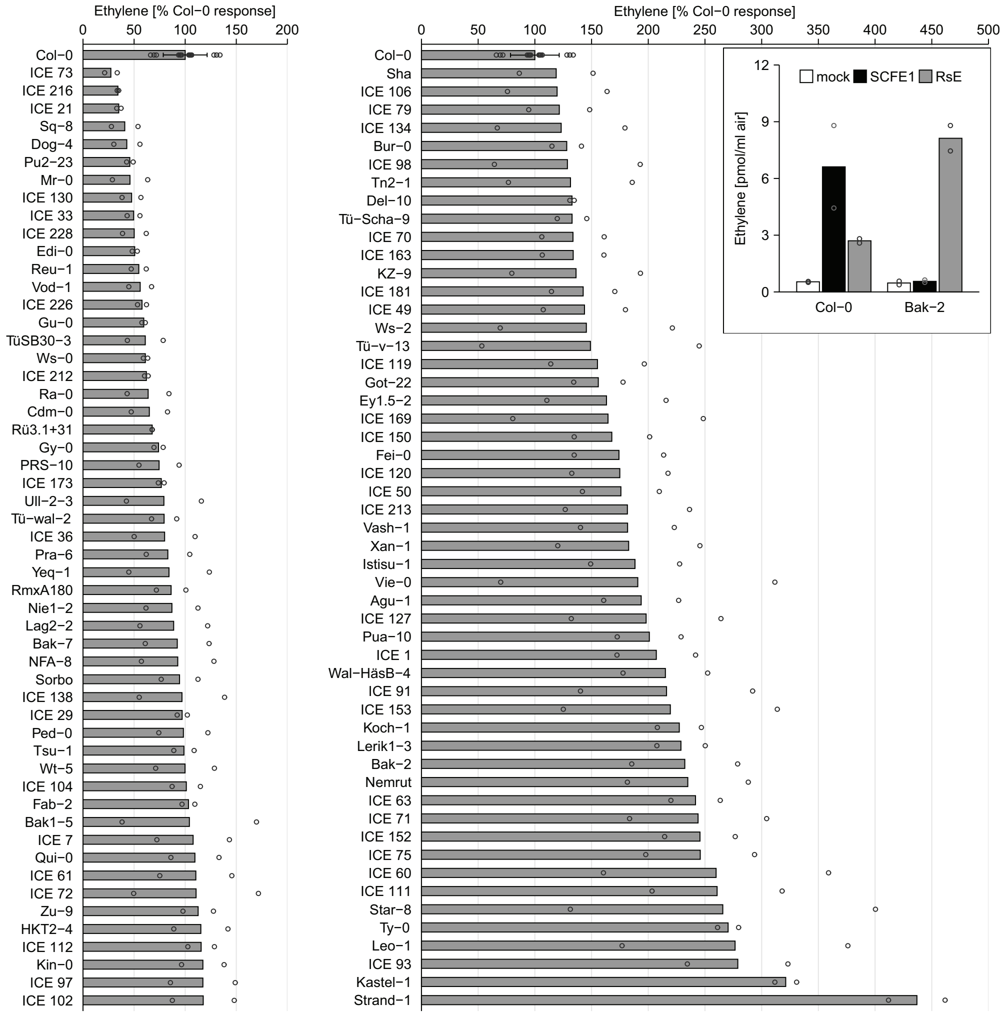

Supplementary Figure 3. **Natural variation in RsE sensitivity among *Arabidopsis* accessions.** 106 *Arabidopsis* accessions were tested for ethylene accumulation upon RsE treatment. Results are shown as percentage of the response determined in Col-0. Bars represent means of two replicates ± SD. Data are pooled from eight experiments. The screen was performed once; accessions used for additional study (indicated by asterisks) were verified in experiments shown in Fig. 1 and Supplementary Figures. 5 and 6. Ethylene production in *Arabidopsis* Col-0 and Bak-2 plants treated with SCFE1 or RsE (inset). Water treatment (mock) served as control. Data points indicate two replicates.

♂ \ ♀	Dog-4	ICE21	ICE73	ICE153
Dog-4	-	y	y	y
ICE21	y	-	y	y
ICE73	n	y	-	y
ICE153	n	y	y	-

Supplementary Figure 4. **Summary of crosses generated for allelism tests.** Shown are *Arabidopsis* accessions used for reciprocal crosses between RsE-insensitive (Dog-4, ICE21, ICE73) and RsE-sensitive (ICE153) accessions. “y” indicates that a normal amount of seeds was obtained; “n” indicates failed crosses, “-“ indicates crosses not performed.

a

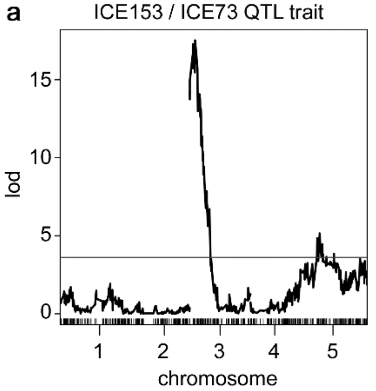

b

Chr	position	LOD	Chr	position	LOD
2	18857542	0,3023032	3	3984300	19,2518605
2	18858222	0,65408905	3	4005543	20,3408255
2	18860115	0,63912253	3	4114238	18,4808285
2	19500124	0,36913299	3	4238450	18,4739649
2	19511097	0,16177605	3	4303467	20,1206988
2	19560723	0,35482815	3	4326887	19,2398194
3	20373	23,7161535	3	4415268	19,7751107
3	96618	20,3589304	3	4426951	18,3413229
3	97041	22,1412548	3	4458826	19,3354048
3	99998	22,2494445	3	4786003	17,1964815
3	100022	21,9456481	3	4786104	18,1941321
3	100053	23,1077577	3	4942846	18,8041414
3	1282003	24,0596519	3	5066042	15,7032338
3	1286337	27,1186078	3	5091471	15,594859
3	1286394	24,1720452	3	5168516	18,0124631
3	1321901	23,0619188	3	5225970	16,686886
3	1432648	26,1204765	3	5268500	12,7434904
3	1546715	26,6332744	3	5533254	15,2878901
3	1784868	25,4141172	3	5666710	13,7562499
3	1784883	25,4103308	3	5666736	13,7562499
3	1784886	25,4107724	3	5940140	11,5326217
3	2012983	25,010172	3	6169544	10,4838362
3	2063924	25,7413858	3	6169557	10,3794269
3	2097697	25,9038471	3	6208231	11,0247178
3	2795414	26,9539864	3	6582498	11,699298
3	3004962	23,5326429	3	6612203	9,29420715
3	3012844	23,7849338	3	6612208	9,29407201
3	3012876	23,7849338	3	6612223	9,29437913
3	3178197	20,2673228	3	6612255	9,49974994
3	3248222	20,8794091	3	6660685	9,38376536
3	3326972	20,0666037	3	6702174	9,64217411
3	3348485	20,8196009	3	6801332	9,0135602
3	3419062	20,4317097	3	7009733	7,48710839
3	3833444	23,0828747	3	7437740	9,07698364
3	3960217	19,6388366	3	7617984	8,83989564

Supplementary Figure 5. **QTL mapping of an RsE-sensitive locus. a** QTL mapping for RsE-induced ethylene response in F_2 mapping populations of an ICE153 x ICE73 cross. LOD scores from a full genome scan across five chromosomes of *Arabidopsis* using a QTL trait model for RsE-elicited ethylene scores were plotted. The horizontal line indicates the significance threshold (an expectation-maximization (EM) algorithm was applied with a permutation of 1,000 repeats and type I error rate alpha =0.05). **b** genomic positions and LOD values derived from QTL. Given are chromosome number (Chr), position on the chromosome and LOD values. Genomic positions with LOD values above 10 based on QTL binary trait mapping (see also Figure 1d) are highlighted in grey.

a

Plate 1	01	02	03	04	05	06	07	08	09	10	11	12
**P1A**	0	0	1	1	1	1	1	1	1	1	1	0
**P1B**	1	1	0	1	1	1	1	1	1	1	0	0
**P1C**	1	1	1	1	1	0	0	1	1	1	1	1
**P1D**	0	1	1	0	1	1	1	1	0	1	1	0
**P1E**	0	1	1	1	1	1	0	1	0	0	0	0
**P1F**	0	0	1	0	0	0	1	0	1	0	1	0
**P1G**	0	0	1	0	1	1	0	1	0	1	0	0
**P1H**	0	0	1	0	0	1	0	0	0	1	0	0

Plate 2	01	02	03	04	05	06	07	08	09	10	11	12
**P2A**	0	0	0	1	0	0	1	1	0	0	1	1
**P2B**	0	0	1	0	0	1	0	1	0	0	0	0
**P2C**	0	1	0	0	0	0	0	0	0	0	0	0
**P2D**	1	0	1	0	0	1	0	0	1	0	0	0
**P2E**	1	1	0	0	0	0	1	0	0	1	0	0
**P2F**	0	1	0	0	0	0	0	1	0	0	0	1
**P2G**	1	1	1	0	0	1	0	0	1	1	1	0
**P2H**	1	0	0	1	0	0	0	0	0	1	1	0

b

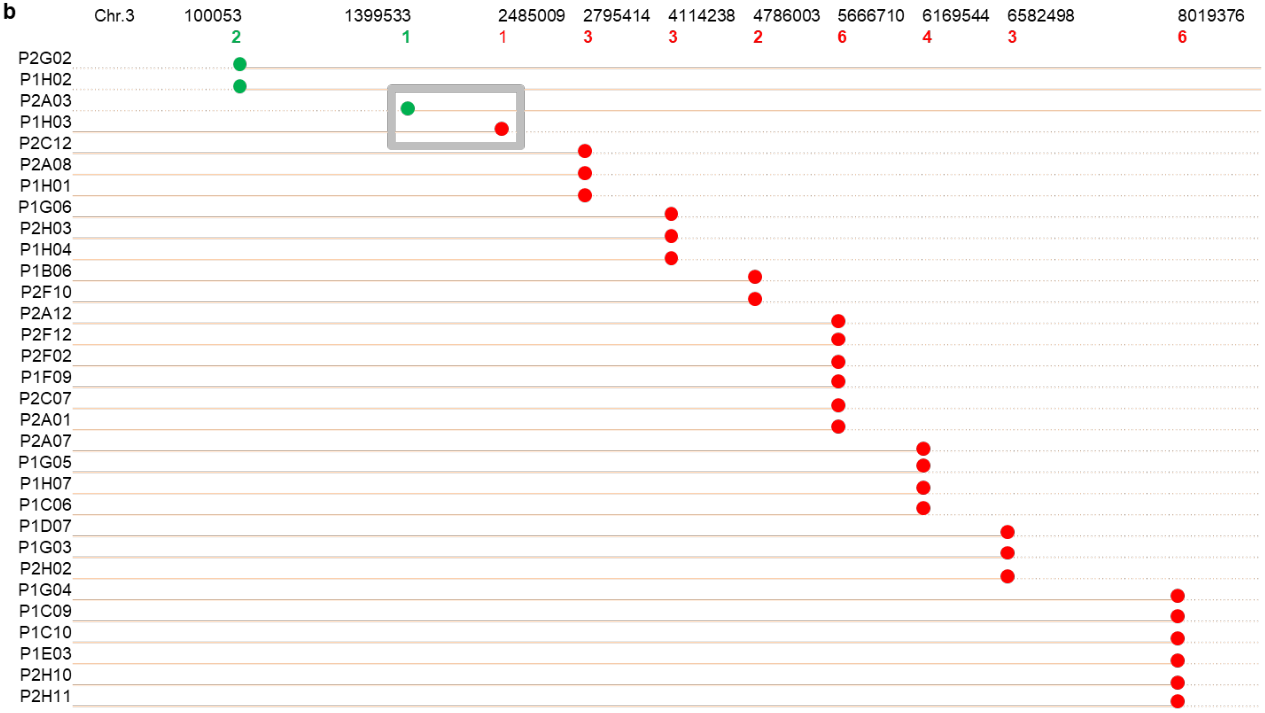

Supplementary Figure 6. **Definition of left and right boundaries of the QTL for RsE sensitivity. a** Binary phenotype layout for the RAD-seq library. The layout was designed according to a 96-well format, each well shows the value 1 (RsE-insensitive) or 0 (RsE-sensitive) of the RsE-induced ethylene response of individual F_2 plants of an ICE153 x ICE73 cross. Insensitive phenotypes are highlighted in grey. **b** Diagram of 31 individual F_2 plants (code given on the left) containing informative recombination events with their genomic positions on chromosome 3 indicated on top. Green numbers indicate summarized frequency of recombination events occurring at the left boundary, red numbers indicate summarized frequency of recombination events occurring at the right boundary within the F_2 population. The grey box represents the QTL-mapping region that is associated with RsE-induced ethylene responses.

Line name	NASC stock number	Allele name	T-DNA position as verified by sequencing
SM_3_33092	N119803	*rlp32-2*	12 bp downstream of start codon
SALK_137467C	N657024	*rlp32-3*	408 bp upstream of start codon
SM_3_33695	N120406	*rlp32-4*	2 bp downstream of start codon
SM3_15851	N106446	*rlp32-5*	487 bp downstream of start codon

Supplementary Figure 7. ***Rlp32* mutant genotypes used in this study.** The position of the T-DNA or transposon insertion was verified in each line by flanking fragment sequencing. The mutant line FLAG_588C11 in the Ws-0 accession was published by Wang *et al.*[1] as *rlp32-1* and was not used in our studies.

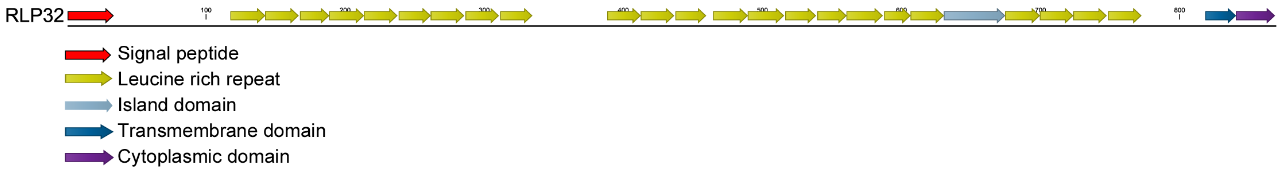

Supplementary Figure 8. **Schematic representation of the RLP32 protein structure.** RLP32 consists of a signal peptide, 23 LRR domains, an island domain, a transmembrane domain and a short cytoplasmic tail. Protein domains identified by UniProt database are indicated in colors.

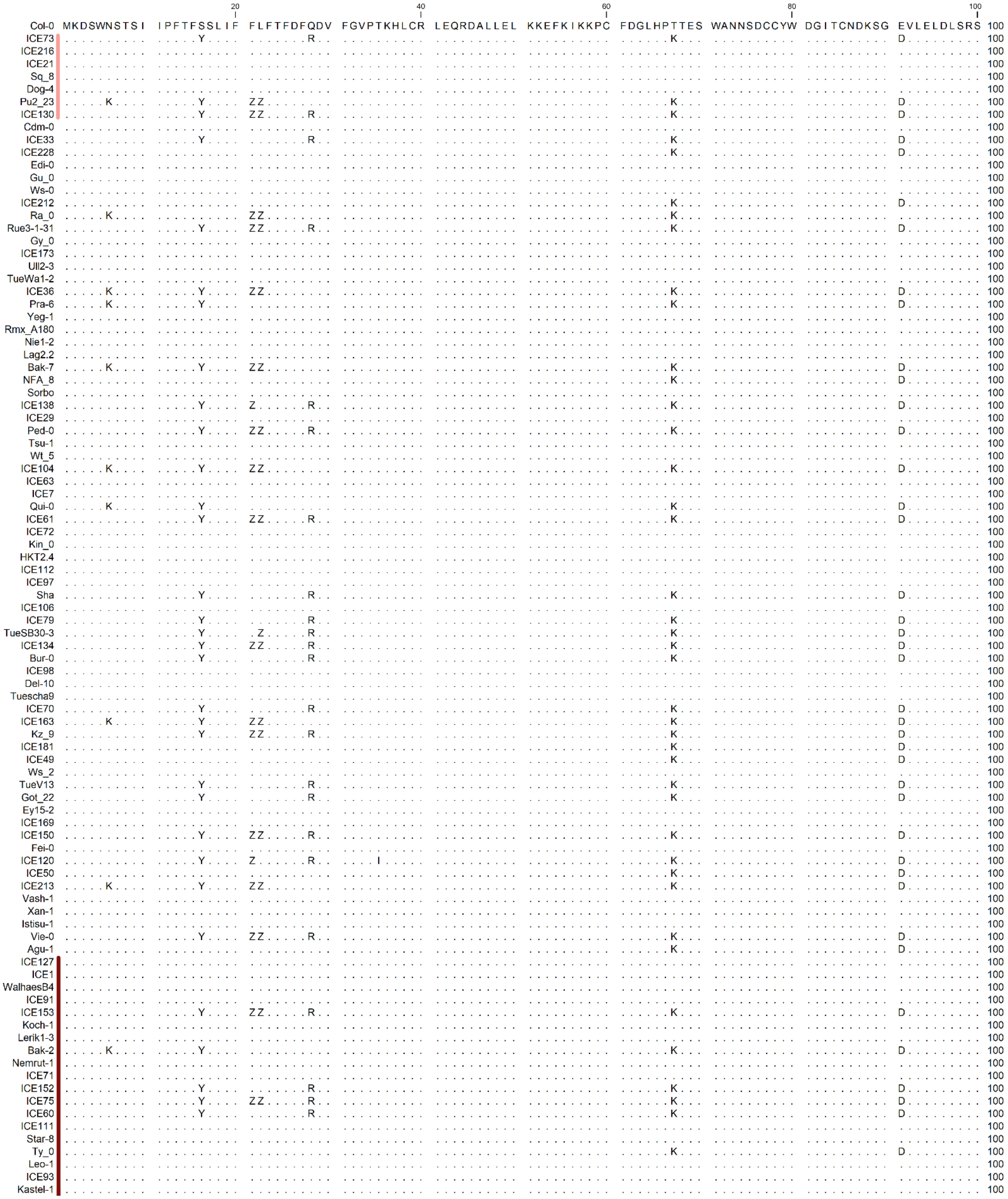
20 40 60 80 100
Col-0 MKDSWNSTSI IPFTFSSLIF FLFTFDFQDV FGVPTKHLCR LEQRDALLEL KKEFKIKKPC FDGLHPTTES WANNSDCCYW DGITCNDKSG EVLELDLSRS 100

```
                                     120                             140                             160                             180                             200
Col-0      CLQSRFHSNS SLFTVLNLRF LTTLDLSYNY FSGQIPSCIE NFSHLTTLDL SKNYFSGGIP SSIGNLSQLT FLDLSGNEFV GEMPFFGNMN QLTNLYVDSN 200
ICE73      .......... ...R.Q..P. .......... .......S.. .......... .......... .......... .......D.. .......T.. .......... 200
ICE216     .......... .....Q.... .......... .......... .......... .......... .......... .......... .......... .......... 200
ICE21      .......... .....Q.... .......... .......... .......... .......... .......... .......... .......... .......... 200
Sq_8       .......... .......... .......... .......... .......... .......... .......... .......... .......... .......... 200
Dog-4      .......... .....Q.... .......... .......... .......... .......... .......... .......... .......... .......... 200
Pu2_23     .......... ...R.Q..H. .......... .......S.. .......... .......... .......... .......D.. .......T.. .......... 200
ICE130     .......... ...R.Q..P. .......... .......S.. .......... .......... .......... .......D.. .......T.. .......... 200
Cdm-0      .......... .....Q.... .......... .......... .......... .......... .......... .......... .......... .......... 200
ICE33      .......... ...R.Q..P. .......... .......S.. .......... .......... .......... .......D.. .......T.. .......... 200
ICE228     .......... ...R.Q..P. .......... .......S.. .......... .......... .......... .......D.. .......T.. .......... 200
Edi-0      .......... .......... .......... .......... .......... .......... .......... .......... .......... .......... 200
Gu_0       .......... .......... .......... .......... .......... .......... .......... .......... .......... .......... 200
Ws-0       .......... .....Q.... .......... .......... .......... .......... .......... .......... .......... .......... 200
ICE212     .......... ...R.Q..P. .......... .......S.. .......... .......... .......... .......D.. .......T.. .......... 200
Ra_0       .......... ...R.Q.... .......... .......S.. .......... .......... .......... .......D.. .......T.. .......... 200
Rue3-1-31  .......... ...R.Q..P. .......... .......S.. .......... .......... .......... .......D.. .......T.. .......... 200
Gy_0       .......... .....Q.... .......... .......... .......... .......... .......... .......... .......... .......... 200
ICE173     .......... .......... .......... .......... .......... .......... .......... .......... .......... .......... 200
Ull2-3     .......... .....Q.... .......... .......... .......... .......... .......... .......... .......... .......... 200
TueWa1-2   .......... .....Q.... .......... .......... .......... .......... .......... .......... .......... .......... 200
ICE36      .......... ...R.Q..H. .......... .......S.. .......... .......... .......... .......D.. .......T.. .......... 200
Pra-6      .......... ...R.Q..H. .......... ......YS.. .......... .......... .......... .......D.. .......T.. .......... 200
Yeg-1      Y......... .....Q.... .......... .......... .......... .......... .......... .......... .......... .......... 200
Rmx_A180   .......... .....Q.... .......... .......... .......... .......... .......... .......... .......... .......... 200
Nie1-2     .......... .......... .......... .......... .......... .......... .......... .......... .......... .......... 200
Lag2.2     .......... .....Q.... .......... .......... .......... .......... .......... .......... .......... .......... 200
Bak-7      .......... ...R.Q..H. .......... .......S.. .......... .......... .......... .......D.. .......T.. .......... 200
NFA_8      .......... ...R.Q..P. .......... .......S.. .......... .......... .......... .......D.. .......T.. .......... 200
Sorbo      .......... .....Q.... .......... .......... .......... .......... .......... .......... .......... .......... 200
ICE138     .......... ...R.Q..P. .......... .......S.. .......... .......... .......... .......D.. .......T.. .......... 200
ICE29      .......... .....Q.... .......... .......... .......... .......... .......... .......... .......... .......... 200
Ped-0      ..K....... ...R.Q..H. .......... .......S.. .......... .......... .......... .......D.. .......T.. .......... 200
Tsu-1      .......... .......... .......... .......... .......... .......... .......... .......... .......... .......... 200
Wt_5       .......... .......... .......... .......... .......... .......... .......... .......... .......... .......... 200
ICE104     .......... ...R.Q..H. .......... .......S.. .......... .......... .......... .......D.. .......T.. .......... 200
ICE63      .......... .....Q.... .......... .......... .......... .......... .......... .......... .......... .......... 200
ICE7       .......... .....Q.... .......... .......... .......... .......... .......... .......... .......... .......... 200
Qui-0      .......... ...R.Q..H. .......... .......S.. .......... .......... .......... .......D.. .......T.. .......... 200
ICE61      .......... ...R.Q..P. .......... .......S.. .......... .......... .......... .......D.. .......T.. .......... 200
ICE72      .......... .....Q.... .......... .......... .......... .......... .......... .......... .......... .......... 200
Kin_0      .......... .....Q.... .......... .......... .......... .......... .......... .......... .......... .......... 200
HKT2.4     .......... .......... .......... .......... .......... .......... .......... .......... .......... .......... 200
ICE112     .......... .....Q.... .......... .......... .......... .......... .......... .......... .......... .......... 200
ICE97      .......... .....Q.... .......... .......... .......... .......... .......... .......... .......... .......... 200
Sha        .......... ...R.Q..P. .......... .......S.. .......... .......... .......... .......D.. .......T.. .......... 200
ICE106     .......... .....Q.... .......... .......... .......... .......... .......... .......... .......... .......... 200
ICE79      .......... ...R.Q..P. .......... .......S.. .......... .......... .......... .......D.. .......T.. .......... 200
TueSB30-3  .......... ...R.Q..P. .......... .......S.. .......... .......... .......... .......D.. .......T.. .......... 200
ICE134     .......... ...R.Q..P. .......... .......S.. .......... .......... .......... .......D.. .......T.. .......... 200
Bur-0      .......... ...R.Q..P. .......... .......S.. .......... .......... .......... .......D.. .......T.. .......... 200
ICE98      .......... .....Q.... .......... .......... .......... .......... .......... .......... .......... .......... 200
Del-10     .......... .....Q.... .......... .......... .......... .......... .......... .......... .......... .......... 200
Tuescha9   .......... .....Q.... .......... .......... .......... .......... .......... .......... .......... .......... 200
ICE70      .......... ...R.Q..P. .......... .......S.. .......... .......... .......... .......D.. .......T.. .......... 200
ICE163     .......... ...R.Q..H. .......... .......S.. .......... .......... .......... .......D.. .......T.. .......... 200
Kz_9       .......... ...R.Q..P. .......... .......S.. .......... .......... .......... .......D.. .......T.. .......... 200
ICE181     .......... ...R.Q..P. .......... .......S.. .......... .......... .......... .......D.. .......T.. .......... 200
ICE49      .......... ...R.Q..P. .......... .......S.. .......... .......... .......... .......D.. .......T.. .......... 200
Ws_2       .......... .....Q.... .......... .......... .......... .......... .......... .......... .......... .......... 200
TueV13     .......... ...R.Q..P. .......... .......S.. .......... .......... .......... .......D.. .......T.. .......... 200
Got_22     .......... ...R.Q..P. .......... .......S.. .......... .......... .......... .......D.. .......T.. .......... 200
Ey15-2     .......... .....Q.... .......... .......... .......... .......... .......... .......... .......... .......... 200
ICE169     .......... .......... .......... .......... .......... .......... .......... .......... .......... .......... 200
ICE150     .......... ...R.Q..P. .......... .......S.. .......... .......... .......... .......D.. .......T.. .......... 200
Fei-0      .......... .....Q.... .......... .......... .......... .......... .......... .......... .......... .......... 200
ICE120     .......... ...R.Q..P. .......... .......... .......... .......... .......... .......... .......... .......... 200
ICE50      .......... ...R.Q..P. .......... .......S.. .......... .......... .......... .......D.. .......T.. .......... 200
ICE213     .......... ...R.Q..H. .......... .......S.. .......... .......... .......... .......D.. .......T.. .......... 200
Vash-1     .......... .....Q.... .......... .......... .......... .......... .......... .......... .......... .......... 200
Xan-1      .......... .....Q.... .......... .......... .......... .......... .......... .......... .......... .......... 200
Istisu-1   .......... .....Q.... .......... .......... .......... .......... .......... .......... .......... .......... 200
Vie-0      .......... ...R.Q..P. .......... .......S.. .......... .......... .......... .......D.. .......T.. .......... 200
Agu-1      .......... ...R.Q..P. .......... .......S.. .......... .......... .......... .......D.. .......T.. .......... 200
ICE127     .......... .....Q.... .......... .......... .......... .......... .......... .......... .......... .......... 200
ICE1       .......... .....Q.... .......... .......... .......... .......... .......... .......... .......... .......... 200
WalhaesB4  .......... .....Q.... .......... .......... .......... .......... .......... .......... .......... .......... 200
ICE91      .......... .....Q.... .......... .......... .......... .......... .......... .......... .......... .......... 200
ICE153     .......... ...R.Q..P. .......... .......S.. .......... .......... .......... .......D.. .......T.. .......... 200
Koch-1     .......... .....Q.... .......... .......... .......... .......... .......... .......... .......... .......... 200
Lerik1-3   .......... .....Q.... .......... .......... .......... .......... .......... .......... .......... .......... 200
Bak-2      .......... ...R.Q..H. .......... .......S.. .......... .......... .......... .......D.. .......T.. .......... 200
Nemrut-1   .......... .....Q.... .......... .......... .......... .......... .......... .......... .......... .......... 200
ICE71      .......... .....Q.... .......... .......... .......... .......... .......... .......... .......... .......... 200
ICE152     .......... ...R.Q..P. .......... .......S.. .......... .......... .......... .......D.. .......T.. .......... 200
ICE75      .......... ...R.Q..P. .......... .......S.. .......... .......... .......... .......D.. .......T.. .......... 200
ICE60      .......... ...R.Q..P. .......... .......S.. .......... .......... .......... .......D.. .......T.. .......... 200
ICE111     .......... .....Q.... .......... .......... .......... .......... .......... .......... .......... .......... 200
Star-8     .......... .......... .......... .......... .......... .......... .......... .......... .......... .......... 200
Ty_0       .......... ...R.Q..P. .......... .......S.. .......... .......... .......... .......D.. .......T.. .......... 200
Leo-1      .......... .....Q.... .......... .......... .......... .......... .......... .......... .......... .......... 200
ICE93      .......... .....Q.... .......... .......... .......... .......... .......... .......... .......... .......... 200
Kastel-1   .......... .....Q.... .......... .......... .......... .......... .......... .......... .......... .......... 200
```

```
                     220        240        260        280        300
Col-0      DLTGIFPLSL LNLKHLSDLS LSRNQFTGTL PSNMSSLSNL EYFEAWGNAF TGTLPSSLFT IASLTSINLR NNQLNGTLEF GNISSPSTLT VLDISNNNFI 300
ICE73      E......... ....Q..... .......... .......... .......... .......... .P........ ..H....... .......... .......... 300
ICE216     .......... .......... .......... .......... .......... .......... .......... .......... .......... .......... 300
ICE21      .......... .......... .......... .......... .......... .......... .......... .......... .......... .......... 300
Sq_8       .......... .......... .......... .......... .......... .......... .......... .......... .......... .......... 300
Dog-4      .......... .......... .......... .......... .......... .......... .......... .......... .......... .......... 300
Pu2_23     E......... ....Q..... .......... .......... .......... .......... .P........ ..H....... .......... .......... 300
ICE130     E......... ....Q..... .......... .......... .......... .......... .P........ ..H....... .......... .......... 300
Cdm-0      .......... .......... .......... .......... .......... .......... .......... .......... .......... .......... 300
ICE33      E......... ....Q..... .......... .......... .......... .......... .P........ ..H....... .......... .......... 300
ICE228     E......... ....Q..... .......... .......... .......... .......... .P........ ..H....... .......... .......... 300
Edi-0      .......... .......... .......... .......... .......... .......... .......... .......... .......... .......... 300
Gu_0       .......... .......... .......... .......... .......... .......... .......... .......... .......... .......... 300
Ws-0       .......... .......... .......... .......... .......... .......... .......... .......... .......... .......... 300
ICE212     E......... ....Q..... .......... .......... .......... .......... .P........ ..H....... .......... .......... 300
Ra_0       E......... ....Q..... .......... .......... .......... .......... .P........ ..H....... .......... .......... 300
Rue3-1-31  E......... ....Q..... .......... .......... .......... .......... .P........ ..H....... .......... .......... 300
Gy_0       E......... .......... .......... .......... .......... .......... .......... .......... .......... .......... 300
ICE173     .......... .......... .......... .......... .......... .......... .......... .......... .......... .......... 300
Ull2-3     .......... .......... .......... .......... .......... .......... .......... .......... .......... .......... 300
TueWa1-2   .......... .......... .......... .......... .......... .......... .......... .......... .......... .......... 300
ICE36      E......... ....Q..... .......... .......... .......... .......... .P........ ..H....... .......... .......... 300
Pra-6      E......... ....Q..... .......... .......... .......... .......... .P........ ..H....... .......... .......... 300
Yeg-1      .......... .......... .......... .......... .......... .......... .......... .......... .......... .......... 300
Rmx_A180   E......... .......... .......... .......... .......... .......... .......... .......... .......... .......... 300
Nie1-2     .......... .......... .......... .......... .......... .......... .......... .......... .......... .......... 300
Lag2.2     .......... .......... .......... .......... .......... .......... .......... .......... .......... .......... 300
Bak-7      E.......P. ....Q..... .......... .......... .......... .......... .P........ ..H....... .......... .......... 300
NFA_8      E......... ....Q..... .......... .......... .......... .......... .P........ ..H....... .......... .......... 300
Sorbo      ...S...... .......... .......... .......... .......... .......... .......... .......... .......... .......... 300
ICE138     E......... ....Q..... .......... .......... .......... .......... .P........ ..H....... .......... .......... 300
ICE29      .......... .......... .......... .......... .......... .......... .......... .......... .......... .......... 300
Ped-0      E......... ....Q..... .......... .......... .......... .......... .P........ ..H....... .......... .......... 300
Tsu-1      .......... .......... .......... .......... .......... .......... .......... .......... .......... .......... 300
Wt_5       .......... .......... .......... .......... .......... .......... .......... .......... .......... .......... 300
ICE104     E......... ....Q..... .......... .......... .......... .......... .P........ ..H....... .......... .......... 300
ICE63      .......... .......... .......... .......... .......... .......... .......... .......... .......... .......... 300
ICE7       .......... .......... .......... .......... .......... .......... .......... .......... .......... .......... 300
Qui-0      E......... ....X..... .......... .......... .......... .......... .P........ ..H....... .......... .......... 300
ICE61      E......... ....Q..... .......... .......... .......... .......... .P........ ..H....... .......... .......... 300
ICE72      ...S...... .......... .......... .......... .......... .......... .......... .......... .......... .......... 300
Kin_0      E......... .......... .......... .......... .......... .......... .......... .......... .......... .......... 300
HKT2.4     .......... .......... .......... .......... .......... .......... .......... .......... .......... .......... 300
ICE112     .......... .......... .......... .......... .......... .......... .......... .......... .......... .......... 300
ICE97      E......... .......... .......... .......... .......... .......... .......... .......... .......... .......... 300
Sha        E......... ....Q..... .......... .......... .......... .......... .P........ ..H....... .......... .......... 300
ICE106     .......... .......... .......... .......... .......... .......... .......... .......... .......... .......... 300
ICE79      E......... ....Q..... .......... .......... .......... .......... .P........ ..H....... .......... .......... 300
TueSB30-3  E......... ....Q..... .......... .......... .......... .......... .P........ ..H....... .......... .......... 300
ICE134     E......... ....Q..... .......... .......... .......... .......... .P........ ..H....... .......... .......... 300
Bur-0      E......... ....Q..... .......... .......... .......... .......... .P........ ..H....... .......... .......... 300
ICE98      E......... .......... .......... .......... .......... .......... .......... .......... .......... .......... 300
Del-10     .......... .......... .......... .......... .......... .......... .......... .......... .......... .......... 300
Tuescha9   .......... .......... .......... .......... .......... .......... .......... .......... .......... .......... 300
ICE70      E......... ....Q..... .......... .......... .......... .......... .P........ ..H....... .......... .......... 300
ICE163     E......... ....Q..... .......... .......... .......... .......... .P........ ..H....... .......... .......... 300
Kz_9       E......... ....Q..... .......... .......... .......... .......... .P........ ..H....... .......... .......... 300
ICE181     E......... ....Q..... .......... .......... .......... .......... .P........ ..H....... .......... .......... 300
ICE49      E......... ....Q..... .......... .......... .......... .......... .P........ ..H....... .......... .......... 300
Ws_2       E......... .......... .......... .......... .......... .......... .......... .......... .......... .......... 300
TueV13     E......... ....Q..... .......... .......... .......... .......... .P........ ..H....... .......... .......... 300
Got_22     E......... ....Q..... .......... .......... .......... .......... .P........ ..H....... .......... .......... 300
Ey15-2     E......... .......... .......... .......... .......... .......... .......... .......... .......... .......... 300
ICE169     .......... .......... .......... .......... .......... .......... .......... .......... .......... .......... 300
ICE150     E......... ....Q..... .......... .......... .......... .......... .P........ ..H....... .......... .......... 300
Fei-0      E......... .......... .......... .......... .......... .......... .......... .......... .......... .......... 300
ICE120     .......... .......... .......... .......... .......... .......... .......... .......... .......... .......... 300
ICE50      E......... ....Q..... .......... .......... .......... .......... .P........ ..H....... .......... .......... 300
ICE213     E......... ....Q..... .......... .......... .......... .......... .P........ ..H....... .......... .......... 300
Vash-1     .......... .......... .......... .......... .......... .......... .......... .......... .......... .......... 300
Xan-1      .......... .......... .......... .......... .......... .......... .......... .......... .......... .......... 300
Istisu-1   .......... .......... .......... .......... .......... .......... .......... .......... .......... .......... 300
Vie-0      E......... ....Q..... .......... .......... .......... .......... .P........ ..H....... .......... .......... 300
Agu-1      E......... ....Q..... .......... .......... .......... .......... .P........ ..H....... .......... .......... 300
ICE127     .......... .......... .......... .......... .......... .......... .......... .......... .......... .......... 300
ICE1       .......... .......... .......... .......... .......... .......... .......... .......... .......... .......... 300
WalhaesB4  .......... .......... .......... .......... .......... .......... .......... .......... .......... .......... 300
ICE91      E......... .......... .......... .......... .......... .......... .......... .......... .......... .......... 300
ICE153     E......... ....Q..... .......... .......... .......... .......... .P........ ..H....... .......... .......... 300
Koch-1     .......... .......... .......... .......... .......... .......... .......... .......... .......... .......... 300
Lerik1-3   .......... .......... .......... .......... .......... .......... .......... .......... .......... .......... 300
Bak-2      E......... ....Q..... .......... .......... .......... .......... .P........ ..H....... .......... .......... 300
Nemrut-1   .......... .......... .......... .......... .......... .......... .......... .......... .......... .......... 300
ICE71      ...S...... .......... .......... .......... .......... .......... .......... .......... .......... .......... 300
ICE152     E......... ....Q..... .......... .......... .......... .......... .P........ ..H....... .......... .......... 300
ICE75      E......... ....Q..... .......... .......... .......... .......... .P........ ..H....... .......... .......... 300
ICE60      E......... ....Q..... .......... .......... .......... .......... .P........ ..H....... .......... .......... 300
ICE111     .......... .......... .......... .......... .......... .......... .......... .......... .......... .......... 300
Star-8     .......... .......... .......... .......... .......... .......... .......... .......... .......... .......... 300
Ty_0       E......... ....Q..... .......... .......... .......... .......... .P........ ..H....... .......... .......... 300
Leo-1      .......... .......... .......... .......... .......... .......... .......... .......... .......... .......... 300
ICE93      .......... .......... .......... .......... .......... .......... .......... .......... .......... .......... 300
Kastel-1   .......... .......... .......... .......... .......... .......... .......... .......... .......... .......... 300
```

```
                                  320        340        360        380        400
                                    |          |          |          |          |
Col-0      GPIPKSISKF INLQDLDLSH LNTQGPVDFS IFTNLKSLQL LNLSHLNTTT TIDLNALFSS HLNSIYSMDL SGNHVSATTK ISVADHHPTQ LISQLYLSGC 400
ICE73      .......... .......... .......... ..M....... .......... .......... .......... .......... S......... .......... 400
ICE216     .......... .......... .......... .......... .......... .......... .......... .......... .......... .......... 400
ICE21      .......... .......... .......... .......... .......... .......... .......... .......... .......... .......... 400
Sq_8       .......... .......... .......... .......... .......... .......... .......... .......... .......... .......... 400
Dog-4      .......... .......... .......... .......... .......... .......... .......... ........I. .......... .......... 400
Pu2_23     .......... .......... .......... ..M....... .......... .......... .......... .......... S......... .......... 400
ICE130     .......... .......... .......... ..M....... .......... .......... .......... .......... S......... .......... 400
Cdm-0      .......... .......... .......... .......... .......... .......... .......... .......... .......... .......... 400
ICE33      .......... .......... .......... ..M....... .......... .......... .......... .......... S......... .......... 400
ICE228     .......... .......... .......... ..M....... .......... .......... .......... .......... S......... .......... 400
Edi-0      .......... .......... .......... .......... .......... .......... .......... .......... .......... .......... 400
Gu_0       .......... .......... .......... .......... .......... .......... .......... .......... .......... .......... 400
Ws-0       .......... .......... .......... .......... .......... .......... .......... .......... .......... .......... 400
ICE212     .......... .......... .......... ..M....... .......... .......... .......... .......... S......... .......... 400
Ra_0       .......... .......... .......... ..M....... .......... .......... .......... .......... S.......M. .......... 400
Rue3-1-31  .......... .......... .......... ..M....... .......... .......... .......... .......... S......... .......... 400
Gy_0       .......... .......... ...X...... .......... .......... .......... .......... .......... .......... .......... 400
ICE173     .......... .......... .......... .......... .......... .......... .......... .......... .......... .......... 400
Ull2-3     .......... .......... .......... .......... .......... .......... .......... .......... .......... .......... 400
TueWa1-2   .......... .......... .......... .......... .......... .......... .......... .......... .......... .......... 400
ICE36      .......... .......... .......... ..M....... .......... .......... .......... .......... S......... .......... 400
Pra-6      .......... .......... .......... ..M....... .......... .......... .......... .......... S.......M. .......... 400
Yeg-1      .......... .......... .......... .......... .......... .......... .......... ........I. .......... .......... 400
Rmx_A180   .......... .......... .......... .......... .......... .......... .......... .......... .......... .......... 400
Nie1-2     .......... .......... .......... .......... .......... .......... .......... .......... .......... .......... 400
Lag2.2     .......... .......... .......... .......... .......... .......... .......... .......... .......... .......... 400
Bak-7      .......... .......... .......... ..M....... .......... .......... .......... .......... S.......M. .......... 400
NFA_8      .......... .......... .......... ..M....... .......... .......... .......... .......... S......... .......... 400
Sorbo      .......... .......... .......... .......... .......... .......... .......... .......... .......... .......... 400
ICE138     .......... .......... .......... ..M....... .......... .......... .......... .......... S......... .......... 400
ICE29      .......... .......... .......... .......... .......... .......... .......... .......... .......... .......... 400
Ped-0      .......... .......... .......... ..M....... .......... .......... .......... .......... S.......M. .......... 400
Tsu-1      .......... .......... .......... .......... .......... .......... .......... .......... .......... .......... 400
Wt_5       .......... .......... .......... .......... .......... .......... .......... .......... .......... .......... 400
ICE104     .......... .......... .......... ..M....... .......... .......... .......... .......... S......... .......... 400
ICE63      .......... .......... .......... .......... .......... .......... .......... .......... .......... .......... 400
ICE7       .......... .......... .......... .......... .......... .......... .......... .......... .......... .......... 400
Qui-0      .......... .......... .......... ..M....... .......... .......... .......... .......... S.......M. .......... 400
ICE61      .......... .......... .......... ..M....... .......... .......... .......... .......... S......... .......... 400
ICE72      .......... .......... .......... .......... .......... .......... .......... .......... .......... .......... 400
Kin_0      .......... .......... .......... .......... .......... .......... .......... .......... .......... .......... 400
HKT2.4     .......... .......... .......... .......... .......... .......... .......... .......... .......... .......... 400
ICE112     .......... .......... .......... .......... .......... .......... .......... .......... .......... .......... 400
ICE97      .......... .......... .......... .......... .......... .......... .......... .......... .......... .......... 400
Sha        .......... .......... .......... ..M....... .......... .......... .......... .......... S......... .......... 400
ICE106     .......... .......... .......... .......... .......... .......... .......... .......... .......... .......... 400
ICE79      .......... .......... .......... ..M....... .......... .......... .......... .......... S......... .......... 400
TueSB30-3  .......... .......... .......... ..M....... .......... .......... .......... .......... S......... .......... 400
ICE134     .......... .......... .......... ..M....... .......... .......... .......... .......... S......... .......... 400
Bur-0      .......... .......... .......... ..M....... .......... .......... .......... .......... S......... .......... 400
ICE98      .......... .......... .......... .......... .......... .......... .......... .......... .......... .......... 400
Del-10     .......... .......... .......... .......... .......... .......... .......... .......... .......... .......... 400
Tuescha9   .......... .......... .......... .......... .......... .......... .......... .......... .......... .......... 400
ICE70      .......... .......... .......... ..M....... .......... .......... .......... .......... S......... .......... 400
ICE163     .......... .......... .......... ..M....... .......... .......... .......... .......... S......... .......... 400
Kz_9       .......... .......... .......... ..M....... .......... .......... .......... .......... S......... .......... 400
ICE181     .......... .......... .......... ..M....... .......... .......... .......... .......... S......... .......... 400
ICE49      .......... .......... .......... ..M....... .......... .......... .......... .......... S......... .......... 400
Ws_2       .......... .......... .......... .......... .......... .......... .......... .......... .......... .......... 400
TueV13     .......... .......... .......... ..M....... .......... .......... .......... .......... S......... .......... 400
Got_22     .......... .......... .......... ..M....... .......... .......... .......... .......... S......... .......... 400
Ey15-2     .......... .......... .......... .......... .......... .......... .......... .......... .......... .......... 400
ICE169     .......... .......... .......... .......... .......... .......... .......... .......... .......... .......... 400
ICE150     .......... .......... .......... ..M....... .......... .......... .......... .......... S......... .......... 400
Fei-0      .......... .......... .......... .......... .......... .......... .......... .......... .......... .......... 400
ICE120     .......... .......... .......... .......... .......... .......... .......... .......... .......... .......... 400
ICE50      .......... .......... .......... ..M....... .......... .......... .......... .......... S......... .......... 400
ICE213     .......... .......... .......... ..M....... .......... .......... .......... .......... S......... .......... 400
Vash-1     .......... .......... .......... .......... .......... .......... .......... .......... .......... .......... 400
Xan-1      .......... .......... .......... .......... .......... .......... .......... .......... .......... .......... 400
Istisu-1   .......... .......... .......... .......... .......... .......... .......... .......... .......... .......... 400
Vie-0      .......... .......... .......... ..M....... .......... .......... .......... .......... S......... .......... 400
Agu-1      .......... .......... .......... ..M....... .......... .......... .......... .......... S......... .......... 400
ICE127     .......... .......... .......... .......... .......... .......... .......... .......... .......... .......... 400
ICE1       .......... .......... .......... .......... .......... .......... .......... .......... .......... .......... 400
WalhaesB4  .......... .......... .......... .......... .......... .......... .......... .......... .......... .......... 400
ICE91      .......... .......... .......... .......... .......... .......... .......... .......... .......... .......... 400
ICE153     .......... .......... .......... ..M....... .......... .......... .......... .......... S......... .......... 400
Koch-1     .......... .......... .......... .......... .......... .......... .......... .......... .......... .......... 400
Lerik1-3   .......... .......... .......... .......... .......... .......... .......... .......... .......... .......... 400
Bak-2      .......... .......... .......... ..M....... .......... .......... .......... .......... S......... .......... 400
Nemrut-1   .......... .......... .......... .......... .......... .......... .......... .......... .......... .......... 400
ICE71      .......... .......... .......... .......... .......... .......... .......... .......... .......... .......... 400
ICE152     .......... .......... .......... ..M....... .......... .......... .......... .......... S......... .......... 400
ICE75      .......... .......... .......... ..M....... .......... .......... .......... .......... S......... .......... 400
ICE60      .......... .......... .......... ..M....... .......... .......... .......... .......... S......... .......... 400
ICE111     .......... .......... .......... .......... .......... .......... .......... .......... .......... .......... 400
Star-8     .......... .......... .......... .......... .......... .......... .......... .......... .......... .......... 400
Ty_0       .......... .......... .......... ..M....... .......... .......... .......... .......... S......... .......... 400
Leo-1      .......... .......... .......... .......... .......... .......... .......... .......... .......... .......... 400
ICE93      .......... .......... .......... .......... .......... .......... .......... .......... .......... .......... 400
Kastel-1   .......... .......... .......... .......... .......... .......... .......... .......... .......... .......... 400
```

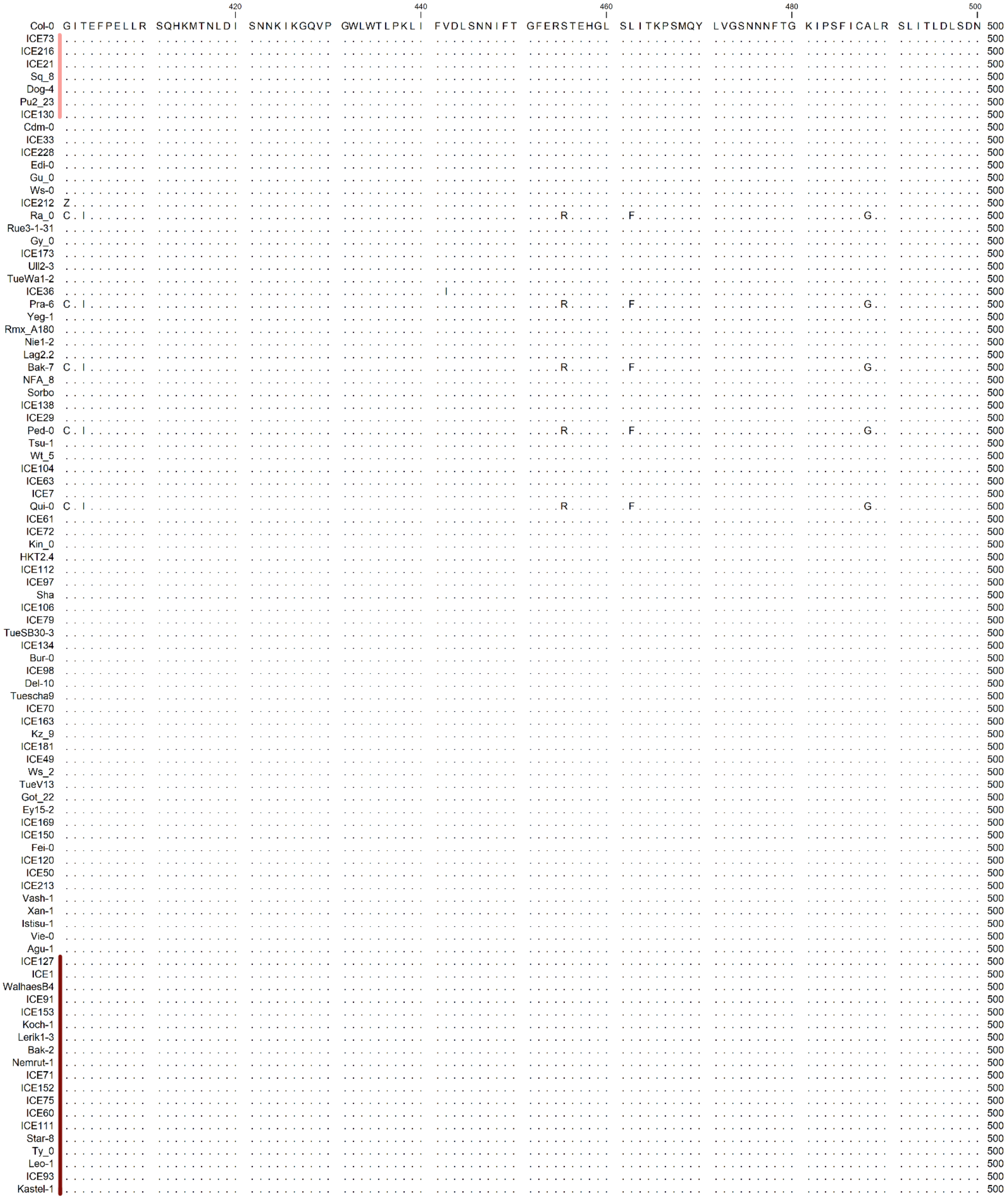

420 440 460 480 500
Col-0 GITEFPELLR SQHKMTNLDI SNNKIKGQVP GWLWTLPKLI FVDLSNNIFT GFERSTEHGL SLITKPSMQY LVGSNNNFTG KIPSFICALR SLITLDLSDN 500
ICE212 Z
Ra_0 C.I R F G
ICE36 I
Pra-6 C.I R F G
Bak-7 C.I R F G
Ped-0 C.I R F G
Qui-0 C.I R F G

```
                                      520                   540                   560                   580                   600
                                        |                     |                     |                     |                     |
Col-0       NLNGSIPPCM GNLKSTLSFL NLRQNRLGGG LPRSIFKSLR SLDVGHNQLV GKLPRSFIRL SALEVLNVEN NRINDTFPFW LSSLKKLQVL VLRSNAFHGP 600
ICE73       .......... .......... .......... .......... .......... .......... .......... .......... .......... .......... 600
ICE216      .......... .......... .......... .......... .......... .......... .......... .......... .......... .......... 600
ICE21       .......... .......... .......... .......... .......... .......... .......... .......... .......... .......... 600
Sq_8        .......... .......... .......... .......... .......... .......... .......... .......... .......... .......... 600
Dog-4       .......... .......... .......... .......... .......... .......... .......... .........X .......... .........A 600
Pu2_23      .......... .......... .......... .......... .......... .......... .......... .......... .......... .......... 600
ICE130      .......... .......... .......... .......... .......... .......... .......... .......... .......... .......... 600
Cdm-0       .......... .......... .......... .......... .......... .......... .......... .......... .......... .......... 600
ICE33       .......... .......... .......... .......... .......... .......... .......... .......... .......... .......... 600
ICE228      .......... .......... .......... .......... .......... .......... .......... .......... .......... .......... 600
Edi-0       .......... .......... .......... .......... .......... .......... .......... .......... .......... .......... 600
Gu_0        .......... .......... .......... .......... .......... .......... .......... .......... .......... .......... 600
Ws-0        .......... .......... .......... .......... .......... .......... .......... .......... .......... .......... 600
ICE212      .......... .......... .......... .......... .......... .......... .......... .......... .......... .......... 600
Ra_0        .......... .......... .......... .......... .......... .......... .......M.. .K........ .......... .......... 600
Rue3-1-31   .......... .......... ...H...... .......... .......... .......... .......... .......... .......... .......... 600
Gy_0        .......... .......... .......... .......... .......... .......... .......... .......... .......... .......... 600
ICE173      .......... .......... .......... .......... .......... .......... .......... .......... .......... .......... 600
Ull2-3      .......... .......... .......... .......... .......... .......... .......... .......... .......... .......... 600
TueWa1-2    .......... .......... .......... .......... .......... .......... .......... .......... .......... .......... 600
ICE36       .......... .......... .......... .......... .......... .......... .......... .......... .......... .......... 600
Pra-6       .......... .......... .......... .......... .......... .......... ....A..M.. .K........ .......... .......... 600
Yeg-1       .......... .......... .......... .......... .......... .......... .......... .......... .......... .........A 600
Rmx_A180    .......... .......... .......... .......... .......... .......... .......... .......... .......... .......... 600
Nie1-2      .......... .......... .......... .......... .......... .......... .......... .......... .......... .......... 600
Lag2.2      .......... .......... .......... .......... .......... .......... .......... .......... .......... .......... 600
Bak-7       .......... .......... .......... .......... .......... .......... ....A..M.. .K........ .......... .......... 600
NFA_8       .......... .......... .......... .......... .......... .......... .......... .......... .......... .......... 600
Sorbo       .......... .......... .......... .......... .......... .......... .......... .......... .......... .......... 600
ICE138      .......... .......... .......... .......... .......... .......... .......... .......... .......... .......... 600
ICE29       .......... .......... .......... .......... .......... .......... .......... .......... .......... .......... 600
Ped-0       .......... .......... .......... .......... .......... .......... ....A..M.. .K........ .......... .......... 600
Tsu-1       .......... .......... .......... .......... .......... .......... .......... .......... .......... .......... 600
Wt_5        .......... .......... .......... .......... .......... .......... .......... .......... .......... .......... 600
ICE104      .......... .......... .......... .......... .......... .......... .......... .......... .......... .......... 600
ICE63       .......... .......... .......... .......... .......... .......... .......... .......... .......... .......... 600
ICE7        .......... .......... .......... .......... .......... .......... .......... .......... .......... .......... 600
Qui-0       .......... .......... .......... .......... .......... .......... ....A..M.. .K........ .......... .......... 600
ICE61       .......... .......... .......... .......... .......... .......... .......... .......... .......... .......... 600
ICE72       .......... .......... .......... .......... .......... .......... .......... .......... .......... .......... 600
Kin_0       .......... .......... .......... .......... .......... .......... .......... .......... .......... .......... 600
HKT2.4      .......... .......... .......... .......... .......... .......... .......... .......... .......... .......... 600
ICE112      .......... .......... .......... .......... .......... .......... .......... .......... .......... .......... 600
ICE97       .......... .......... .......... .......... .......... .......... .......... .......... .......... .......... 600
Sha         .......... .......... .......... .......... .......... .......... .......... .......... .......... .......... 600
ICE106      .......... .......... .......... .......... .......... .......... .......... .......... .......... .......... 600
ICE79       .......... .......... .......... .......... .......... .......... .......... .......... .......... .......... 600
TueSB30-3   .......... .......... .......... .......... .......... .......... .......... .......... .......... .......... 600
ICE134      .......... .......... .......... .......... .......... .......... .......... .......... .......... .......... 600
Bur-0       .......... .......... .......... .......... .......... .......... .......... .......... .......... .......... 600
ICE98       .......... .......... .......... .......... .......... .......... .......... .......... .......... .......... 600
Del-10      .......... .......... .......... .......... .......... .......... .......... .......... .......... .......... 600
Tuescha9    .......... .......... .......... .......... .......... .......... .......... .......... .......... .......... 600
ICE70       .......... .......... .......... .......... .......... .......... .......... .......... .......... .......... 600
ICE163      .......... .......... .......... .......... .......... .......... .......... .......... .......... .......... 600
Kz_9        .......... .......... .......... .......... .......... .......... .......... .......... .......... .......... 600
ICE181      .......... .......... .......... .......... .......... .......... .......... .......... .......... .......... 600
ICE49       .......... .......... .......... .......... .......... .......... .......... .......... .......... .......... 600
Ws_2        .......... .......... .......... .......... .......... .......... .......... .......... .......... .......... 600
TueV13      .......... .......... .......... .......... .......... .......... .......... .......... .......... .......... 600
Got_22      .......... .......... .......... ...G...... .......... .......... .......... .......... .......... .......... 600
Ey15-2      .......... .......... .......... .......... .......... .......... .......... .......... .......... .......... 600
ICE169      .......... .......... .......... .......... .......... .......... .......... .......... .......... .......... 600
ICE150      .......... .......... .......... .......... .......... .......... .......... .......... .......... .......... 600
Fei-0       .......... .......... .......... .......... .......... .......... .......... .......... .......... .......... 600
ICE120      .......... .......... .......... .......... .......... .......... .......... .......... .......... .......... 600
ICE50       .......... .......... .......... .......... .......... .......... .......... .......... .......... .......... 600
ICE213      .......... .......... .......... .......... .......... .......... .......... .......... .......... .......... 600
Vash-1      .......... .......... .......... .......... .......... .......... .......... .......... .......... .......... 600
Xan-1       .......... .......... .......... .......... .......... .......... .......... .......... .......... .......... 600
Istisu-1    .......... .......... .......... .......... .......... .......... .......... .......... .......... .......... 600
Vie-0       .......... .......... .......... .......... .......... .......... .......... .......... .......... .......... 600
Agu-1       .......... .......... .......... .......... .......... .......... .......... .......... .......... .......... 600
ICE127      .......... .......... .......... .......... .......... .......... .......... .......... .......... .........A 600
ICE1        .......... .......... .......... .......... .......... .......... .......... .......... .......... .......... 600
WalhaesB4   .......... .......... .......... .......... .......... .......... .......... .......... .......... .......... 600
ICE91       .......... .......... .......... .......... .......... .......... .......... .......... .......... .......... 600
ICE153      .......... .......... .......... .......... .......... .......... .......... .......... .......... .......... 600
Koch-1      .......... .......... .......... .......... .......... .......... .......... .......... .......... .......... 600
Lerik1-3    .......... .......... .......... .......... .......... .......... .......... .......... .......... .......... 600
Bak-2       .......... .......... .......... .......... .......... .......... .......... .......... .......... .......... 600
Nemrut-1    .......... .......... .......... .......... .......... .......... .......... .......... .......... .........A 600
ICE71       .......... .......... .......... .......... .......... .......... .......... .......... .......... .......... 600
ICE152      .......... .......... .......... .......... .......... .......... .......... .......... .......... .......... 600
ICE75       .......... .......... .......... .......... .......... .......... .......... .......... .......... .......... 600
ICE60       .......... .......... .......... .......... .......... .......... .......... .......... .......... .......... 600
ICE111      .......... .......... .......... .......... .......... .......... .......... .......... .......... .......... 600
Star-8      .......... .......... .......... .......... .......... .......... .......... .......... .......... .......... 600
Ty_0        .......... .......... .......... .......... .......... .......... .......... .......... .......... .......... 600
Leo-1       .......... .......... .......... .......... .......... .......... .......... .......... .......... .......... 600
ICE93       .......... .......... .......... .......... .......... .......... .......... .......... .......... .......... 600
Kastel-1    .......... .......... .......... .......... .......... .......... .......... .......... .......... .......... 600
```

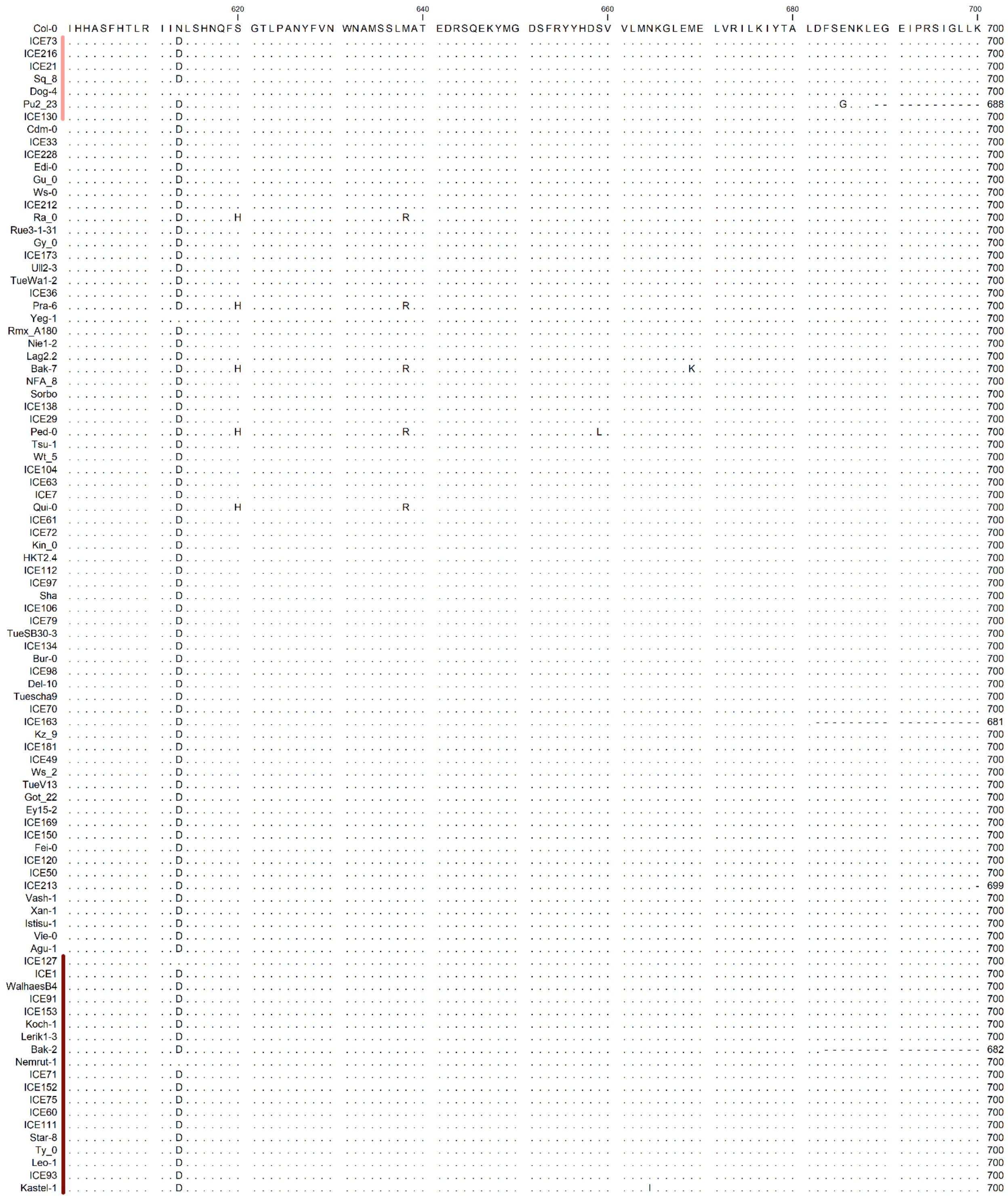
620 640 660 680 700
Col-0 IHHASFHTLR IINLSHNQFS GTLPANYFVN WNAMSSLMAT EDRSQEKYMG DSFRYYHDSV VLMNKGLEME LVRILKIYTA LDFSENKLEG EIPRSIGLLK 700
ICE73 ....D....... 700
ICE216 ....D....... 700
ICE21 ....D....... 700
Sq_8 ....D....... 700
Dog-4 700
Pu2_23 ....D....... ....G...-- ---------- 688
ICE130 ....D....... 700
Cdm-0 ....D....... 700
ICE33 ....D....... 700
ICE228 ....D....... 700
Edi-0 ....D....... 700
Gu_0 ....D....... 700
Ws-0 ....D....... 700
ICE212 ....D....... 700
Ra_0 ....D......H ....R. 700
Rue3-1-31 ....D....... 700
Gy_0 ....D....... 700
ICE173 ....D....... 700
Ull2-3 ....D....... 700
TueWa1-2 ....D....... 700
ICE36 ....D....... 700
Pra-6 ....D......H ....R. 700
Yeg-1 700
Rmx_A180 ....D....... 700
Nie1-2 ....D....... 700
Lag2.2 ....D....... 700
Bak-7 ....D......H ....R. ....K. 700
NFA_8 ....D....... 700
Sorbo ....D....... 700
ICE138 ....D....... 700
ICE29 ....D....... 700
Ped-0 ....D......H ....R. ....L. 700
Tsu-1 ....D....... 700
Wt_5 ....D....... 700
ICE104 ....D....... 700
ICE63 ....D....... 700
ICE7 ....D....... 700
Qui-0 ....D......H ....R. 700
ICE61 ....D....... 700
ICE72 ....D....... 700
Kin_0 ....D....... 700
HKT2.4 ....D....... 700
ICE112 ....D....... 700
ICE97 ....D....... 700
Sha ....D....... 700
ICE106 ....D....... 700
ICE79 ....D....... 700
TueSB30-3 ....D....... 700
ICE134 ....D....... 700
Bur-0 ....D....... 700
ICE98 ....D....... 700
Del-10 ....D....... 700
Tuescha9 ....D....... 700
ICE70 ....D....... 700
ICE163 ....D....... ....--------- ---------- 681
Kz_9 ....D....... 700
ICE181 ....D....... 700
ICE49 ....D....... 700
Ws_2 ....D....... 700
TueV13 ....D....... 700
Got_22 ....D....... 700
Ey15-2 ....D....... 700
ICE169 ....D....... 700
ICE150 ....D....... 700
Fei-0 ....D....... 700
ICE120 ....D....... 700
ICE50 ....D....... 700
ICE213 ....D....... ....- 699
Vash-1 ....D....... 700
Xan-1 ....D....... 700
Istisu-1 ....D....... 700
Vie-0 ....D....... 700
Agu-1 ....D....... 700
ICE127 700
ICE1 ....D....... 700
WalhaesB4 ....D....... 700
ICE91 ....D....... 700
ICE153 ....D....... 700
Koch-1 ....D....... 700
Lerik1-3 ....D....... 700
Bak-2 ....D....... ....-------- ---------- 682
Nemrut-1 700
ICE71 ....D....... 700
ICE152 ....D....... 700
ICE75 ....D....... 700
ICE60 ....D....... 700
ICE111 ....D....... 700
Star-8 ....D....... 700
Ty_0 ....D....... 700
Leo-1 ....D....... 700
ICE93 ....D....... 700
Kastel-1 ....D....... ....I..... 700

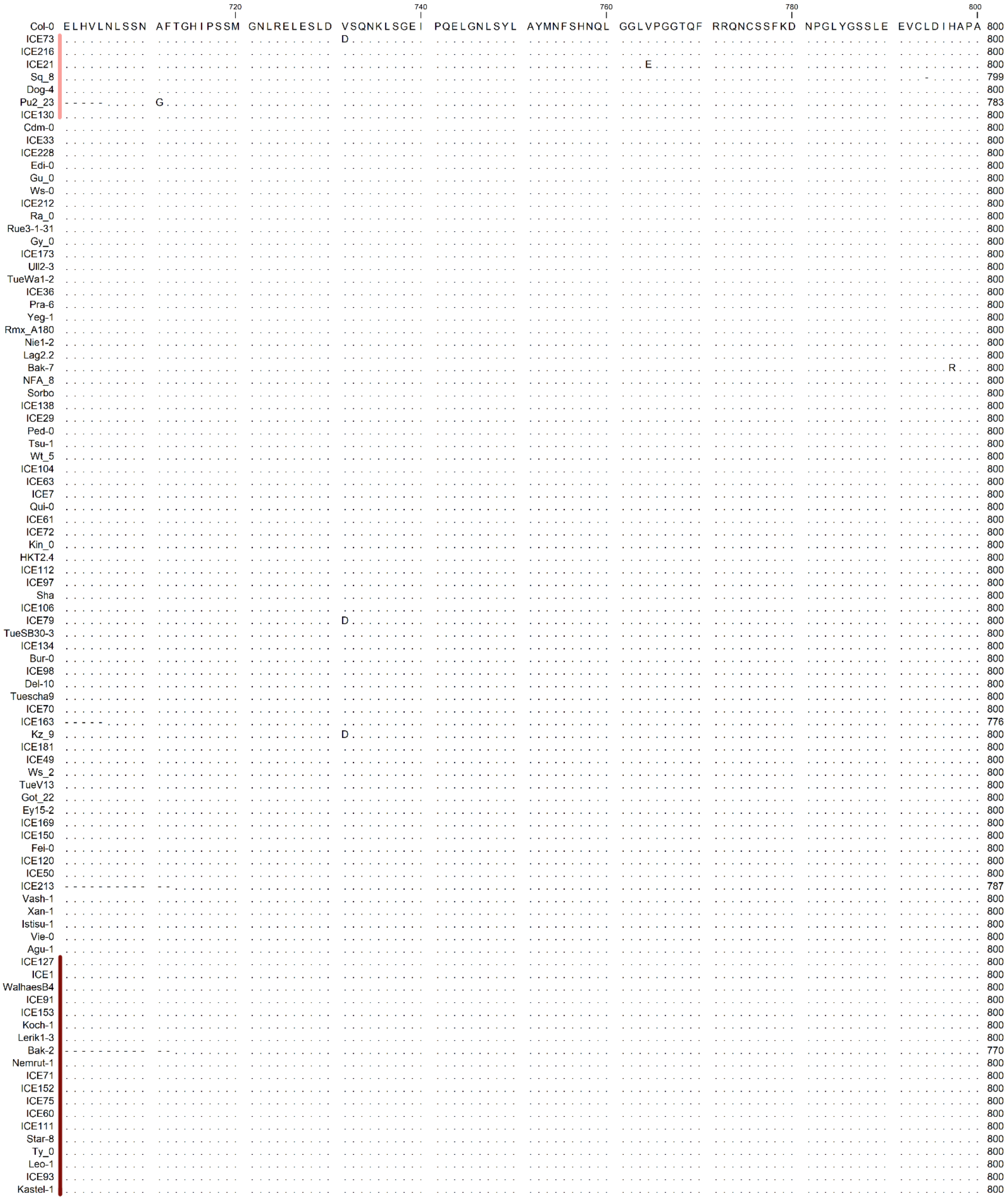
720
740
760
780
800
Col-0 ELHVLNLSSN AFTGHIPSSM GNLRELESLD VSQNKLSGEI PQELGNLSYL AYMNFSHNQL GGLVPGGTQF RRQNCSSFKD NPGLYGSSLE EVCLDIHAPA 800
ICE73 D 800
ICE216 800
ICE21 E 800
Sq_8 - 799
Dog-4 800
Pu2_23 - - - - - G 783
ICE130 800
Cdm-0 800
ICE33 800
ICE228 800
Edi-0 800
Gu_0 800
Ws-0 800
ICE212 800
Ra_0 800
Rue3-1-31 800
Gy_0 800
ICE173 800
Ull2-3 800
TueWa1-2 800
ICE36 800
Pra-6 800
Yeg-1 800
Rmx_A180 800
Nie1-2 800
Lag2.2 800
Bak-7 R 800
NFA_8 800
Sorbo 800
ICE138 800
ICE29 800
Ped-0 800
Tsu-1 800
Wt_5 800
ICE104 800
ICE63 800
ICE7 800
Qui-0 800
ICE61 800
ICE72 800
Kin_0 800
HKT2.4 800
ICE112 800
ICE97 800
Sha 800
ICE106 800
ICE79 D 800
TueSB30-3 800
ICE134 800
Bur-0 800
ICE98 800
Del-10 800
Tuescha9 800
ICE70 800
ICE163 - - - - - 776
Kz_9 D 800
ICE181 800
ICE49 800
Ws_2 800
TueV13 800
Got_22 800
Ey15-2 800
ICE169 800
ICE150 800
Fei-0 800
ICE120 800
ICE50 800
ICE213 - - - - - - - - - - - - 787
Vash-1 800
Xan-1 800
Istisu-1 800
Vie-0 800
Agu-1 800
ICE127 800
ICE1 800
WalhaesB4 800
ICE91 800
ICE153 800
Koch-1 800
Lerik1-3 800
Bak-2 - - - - - - - - - - - - 770
Nemrut-1 800
ICE71 800
ICE152 800
ICE75 800
ICE60 800
ICE111 800
Star-8 800
Ty_0 800
Leo-1 800
ICE93 800
Kastel-1 800

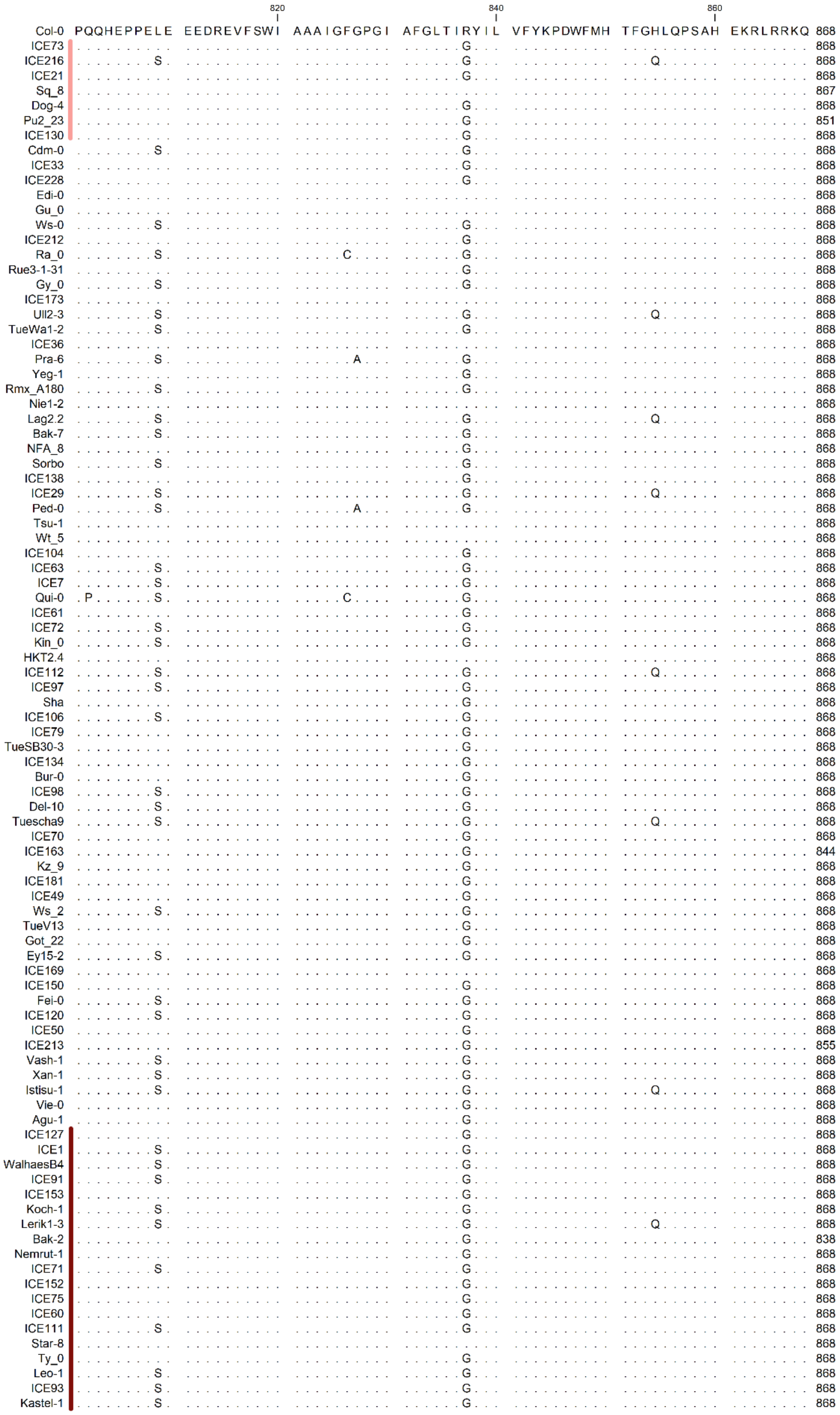

Supplementary Figure 9. **Amino acid sequence alignment of RLP32 from 93 *A. thaliana* accessions.** The accessions are ordered according to the percentage of ethylene response upon RsE treatment determined in Col-0, according to the result from Supplementary Figure 3. RsE-insensitive accessions are marked by pink lines. RsE-hypersensitive accessions (at least 2-fold higher ethylene production than in Col-0) are marked by dark red lines. Matching residues and missing residues are indicated as dots and dashes, respectively. An “X” indicates an amino acid codon has become a stop codon. A “Z” indicates an unknown amino acid.

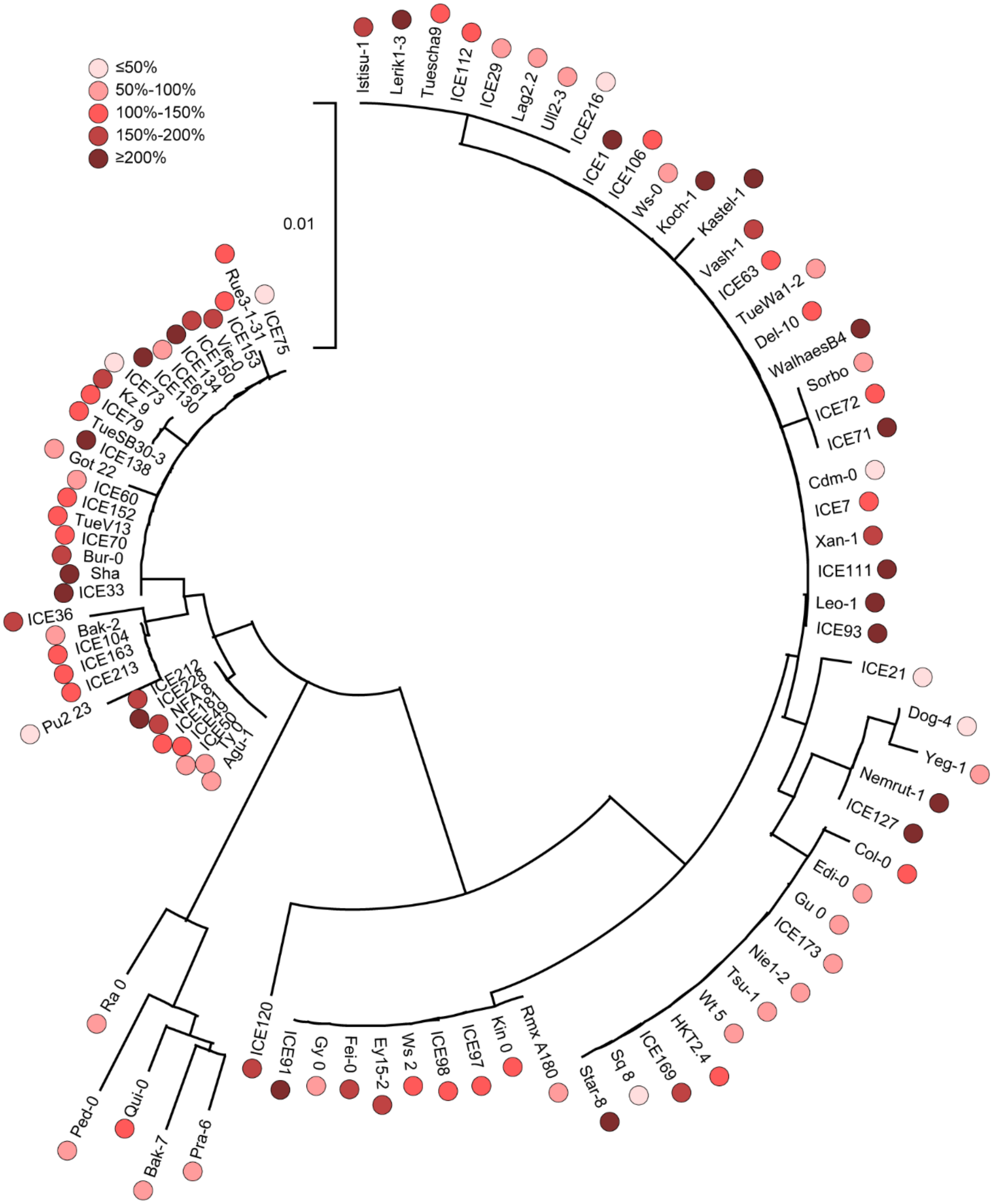

Supplementary Figure 10. **Phylogenetic analysis of RLP32 amino acid sequences from 93 *A. thaliana* accessions.** The evolutionary history was inferred using the Neighbor-Joining method. The optimal tree with the sum of branch length = 0.07613851 is shown. The tree is drawn to scale, with branch lengths in the same units as those of the evolutionary distances used to infer the phylogenetic tree. The evolutionary distances were computed using the Poisson correction method and the evolutionary analyses were conducted in MEGA X (https://www.megasoftware.net/). The percentage of ethylene response upon RsE treatment determined in Col-0, according to the result from Supplementary Figure 3, is divided in 5 grades, with darker colours representing more ethylene production.

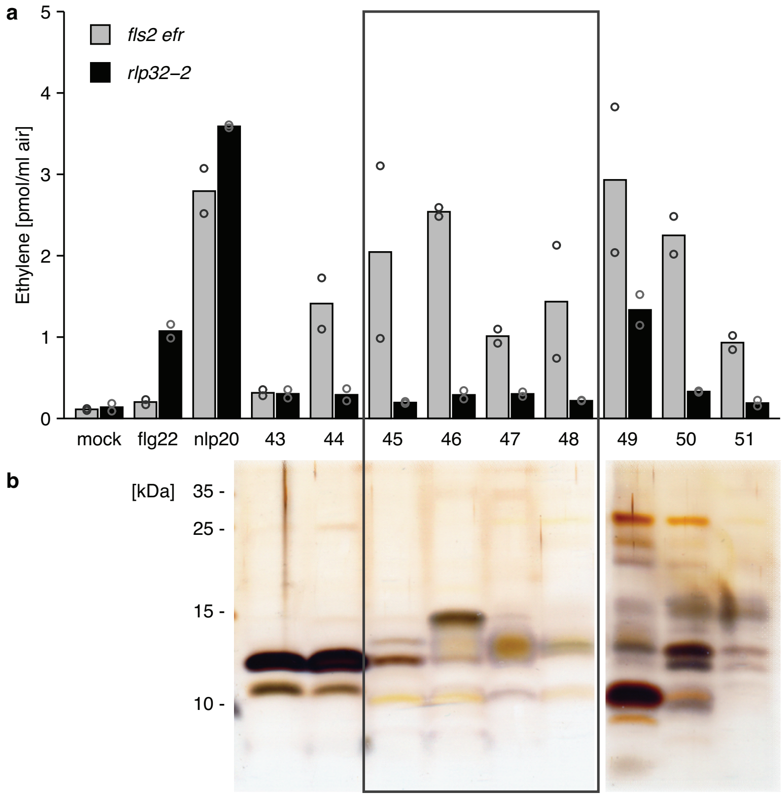

c

	Fraction					
	45	46	47	48		
Protein names	Number of peptides				[kDa]	IP
Uncharacterized protein YibL	15	23	25	17	13.70	9.49
Uncharacterized protein YdcH	17	18	16	14	8.88	9.3
50S ribosomal protein L24	16	13	14	10	11.32	10.21
50S ribosomal protein L22	10	10	13	17	12.23	10.23
50S ribosomal protein L13	13	13	11	12	16.02	9.91
50S ribosomal protein L17	10	8	12	11	14.36	11.05
50S ribosomal protein L6	9	8	13	11	18.90	9.71
50S ribosomal protein L30	9	12	12	6	6.54	10.96
Spheroplast protein Y	10	9	11	7	18.20	9.66
30S ribosomal protein S16	10	9	8	8	9.19	10.54
Uncharacterized protein YciH	9	8	7	10	11.40	9.38
Chaperone protein Skp	7	5	10	12	17.69	9.69
Protein YqjC	9	6	9	9	13.91	8.81
50S ribosomal protein L31	6	6	8	8	7.87	9.46
Translation initiation factor IF-1	6	7	7	7	8.25	9.22
Acylphosphatase	7	6	7	6	10.30	8.66
Met repressor	7	7	7	5	12.14	5.4
50S ribosomal protein L25	3	6	8	9	10.69	9.6
30S ribosomal protein S19	10	7	6	2	10.43	10.52
30S ribosomal protein S20	6	8	5	5	9.68	11.18

Supplementary Figure 11. **Identification of RLP32-dependent elicitor activity in fractionated *E. coli* proteins.** **a** *E. coli* protein fractions separated by C8 reverse phase HPLC were assessed for eliciting ethylene production in *Arabidopsis fls2 efr* or *rlp32* plants. Treatment with water (mock), flg22 or nlp20 served as controls. Data points indicate two replicates. The experiment was performed three times with similar results. **b** Tricine-SDS-PAGE of proteins shown in (**a**). Proteins were visualized by silver staining. Boxed fractions 45-48 representing RLP32-dependent elicitor activity were analyzed by LC-MS/MS. **c** List of proteins identified by LC-MS/MS. Shown are total numbers of peptides representing proteins identified by LC-MS/MS together with molecular masses (kDa) and isoelectric points (IP) of these proteins.

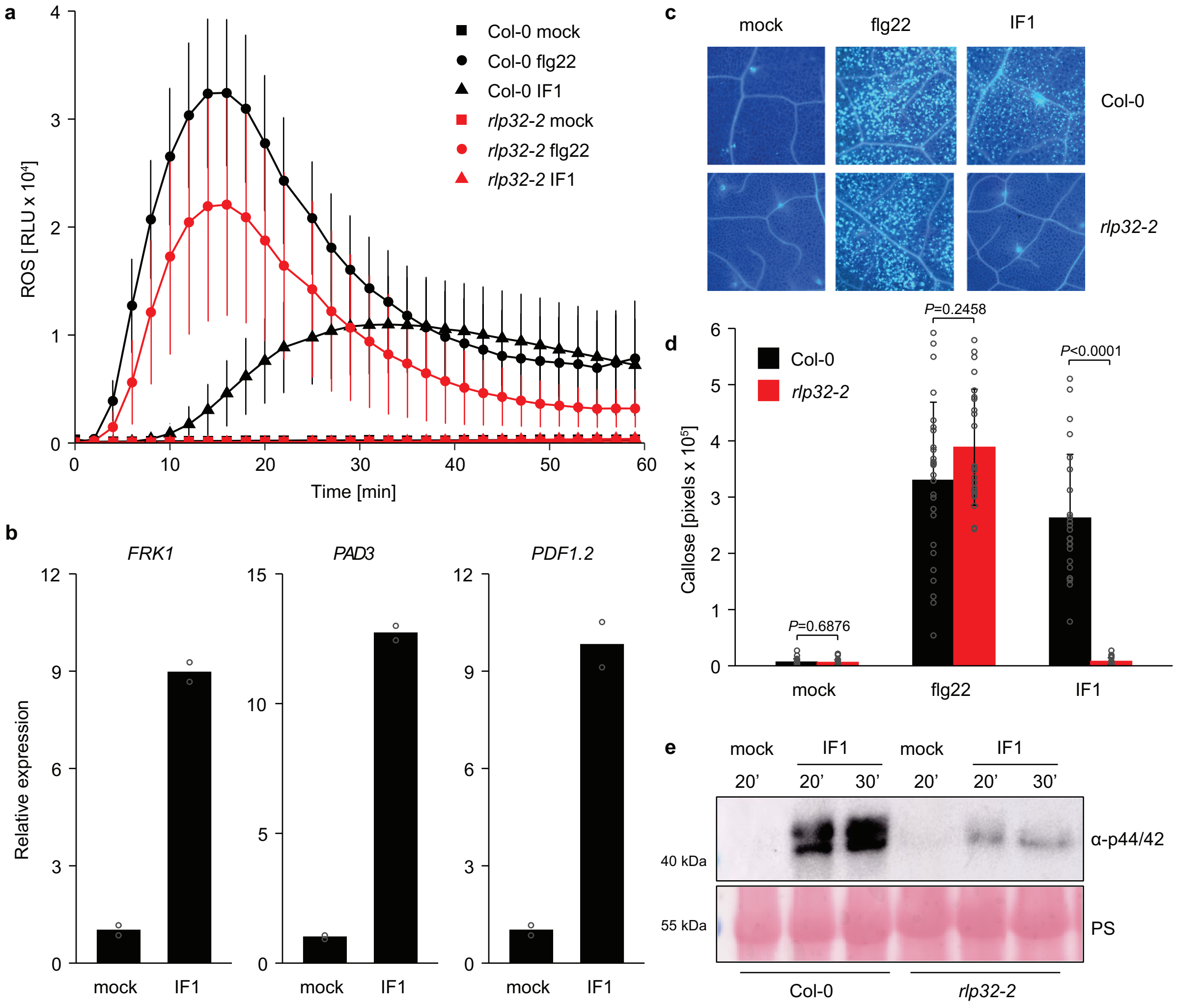

Supplementary Figure 12. **IF1 triggers defense responses in *Arabidopsis*.** **a** ROS accumulation in leaf pieces of *Arabidopsis* Col-0 or *rlp32-2* plants treated with water (mock), 1 µM flg22 or 1 µM IF1. Given are relative light units (RLU) ± SD (*n*=6 for mock and flg22, *n*=12 for IF1). **b** Defense gene expression in leaves of *Arabidopsis* Col-0 plants infiltrated for 24 h with water (mock), or 1 µM IF1. Given is mean expression of the indicated genes relative to mock treatment for two biological replicates. **c** Aniline blue stain of callose appositions 24 h after treatment of Col-0 or *rlp32-2* leaves with water (mock), flg22, or IF1. **d** Quantification of callose apposition shown in (**c**) by ImageJ. Given are mean pixel counts ± SD (for Col-0 treated with flg22 *n*=27, for all others *n*=24. Statistically significant differences in responses between Col-0 and *rlp32-2* were determined using a two-sided Mann-Whitney-U-test. **e** *Arabidopsis* Col-0 or *rlp32-2* plants were treated for the times indicated with water (mock), or 1 µM IF1. MAPK activation was detected by immunoblot using phospho-p44/p42 antibodies. Equal protein loading was verified with staining of the membrane with Ponceau S Red (PS). Experiments were performed at least two times with similar results.

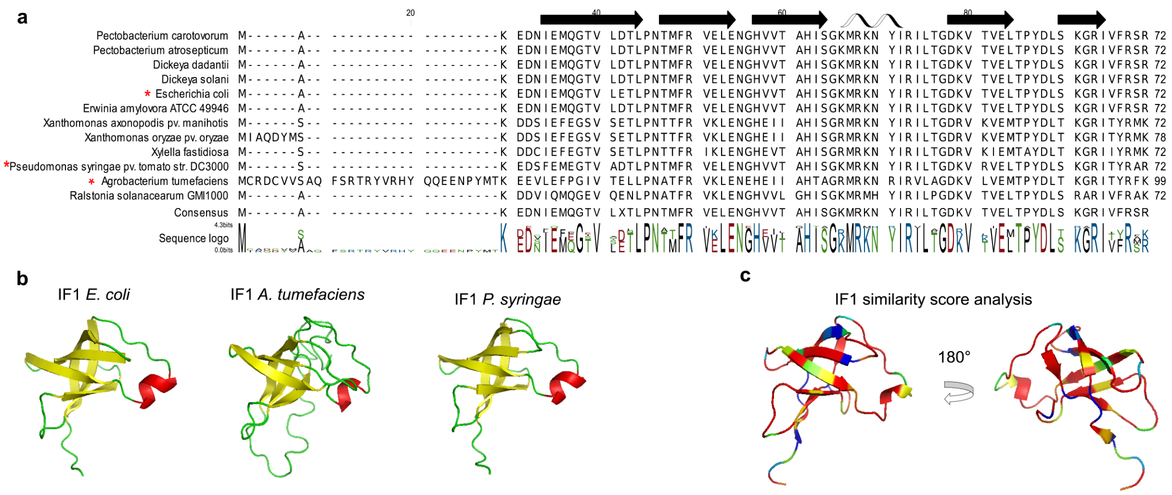

Supplementary Figure 13. **IF1 is conserved in *Proteobacteria*. a** Alignment of IF1 amino acid sequences from different *Proteobacteria* species. Secondary structures (beta sheets indicated as black arrows and alpha helix indicated as grey helix) are indicated atop the protein sequences. Red asterisks indicate organisms from which IF1 was cloned and recombinantly expressed (see also Figure 2d). **b** Iterative threading assembly refinement (I-TASSER) 3D structure prediction of IF1 derived from *E. coli*, *P. syringae* and *A. tumefaciens* as ribbon presentation (yellow indicates beta sheet, red indicates alpha helix). As a template, the NMR structure of IF1 derived from *E. coli* was used[2]. **c** Ribbon representation of IF1, colored by conservation (blue indicates low conservation; red indicates high conservation). Conservation scores for each amino acid were calculated and mapped onto the IF1 structure with Easy Sequencing in PostScript (ESPript 2.2). Ribbon presentation was generated with PYMOL.

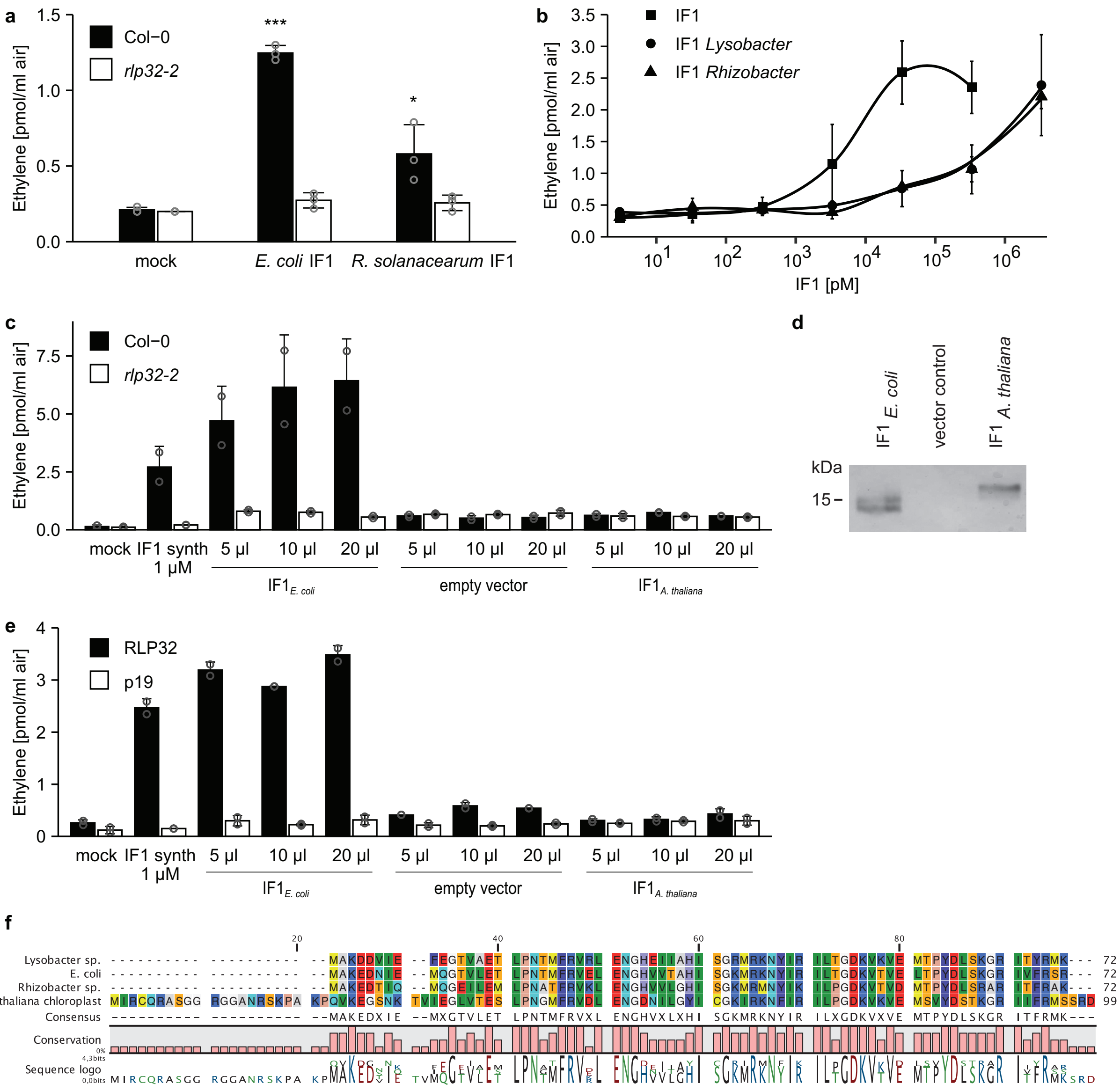

Supplementary Figure 14. **Elicitor activity of microbe- and plant-derived IF1 preparations.** Ethylene accumulation in *Arabidopsis* Col-0 **(a-c)** and *rlp32-2* mutant plants (**a**,**c**) or *N. benthamiana* plants transiently transformed with *Arabidopsis* RLP32 (**e**) after treatment with IF1. **a** Treatment with 1 µM synthetic *E. coli* or *R. solanacearum* IF1, respectively. Water treatment served as control (mock). Bars represent means ± SD of three replicates. Statistically significant differences in responses mock and elicitor treatments were determined using a two-sided Dunnett's test with mock as control. Exact p-values are provided in the Source data file. **b** Treatment with increasing concentrations of synthetic *E. coli* (IF1), or synthetic IF1 from *Lysobacter* spp. or *Rhizobacter* spp. Points and error bars represent means ± SD of three replicates. **c**, **e** Treatment with crude culture filtrates of *Pichia pastoris* expressing recombinant *E. coli* and *Arabidopsis* chloroplast IF1. Treatment with water (mock) or with protein extracts from *P. pastoris* transformed with control plasmid (empty vector) served as controls. Plants transformed with viral gene silencing suppressor p19 only served as negative controls. Data points indicate two replicates. **d** Immunoblot analysis of recombinant IF1 used in **c**, **e**, detected with anti-c-myc antibodies. Gels were loaded with protein precipitated from 200 µl of culture filtrate used in **c**, **e**. Experiments were performed two times with similar results. **f** Alignment of IF1 amino acid sequences from *E. coli, Lysobacter*, *Rhizobacter* and *A. thaliana* Col-0 chloroplast.

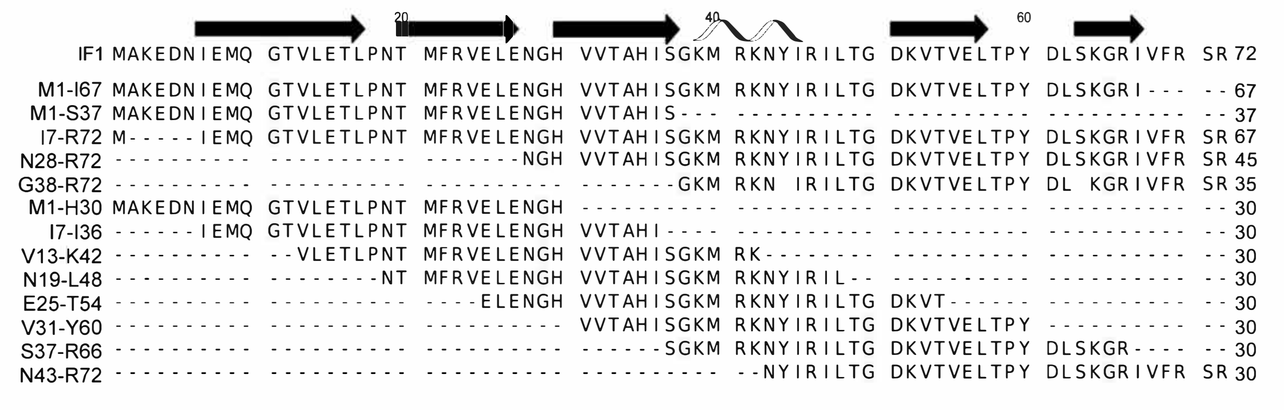

Supplementary Figure 15. **Alignment of amino acid sequences of *E. coli* IF1 deletion constructs and synthetic peptides used in Figure 4a, b**. black arrows (beta sheets) and a helix symbol (alpha helix) indicating IF1 secondary structures.

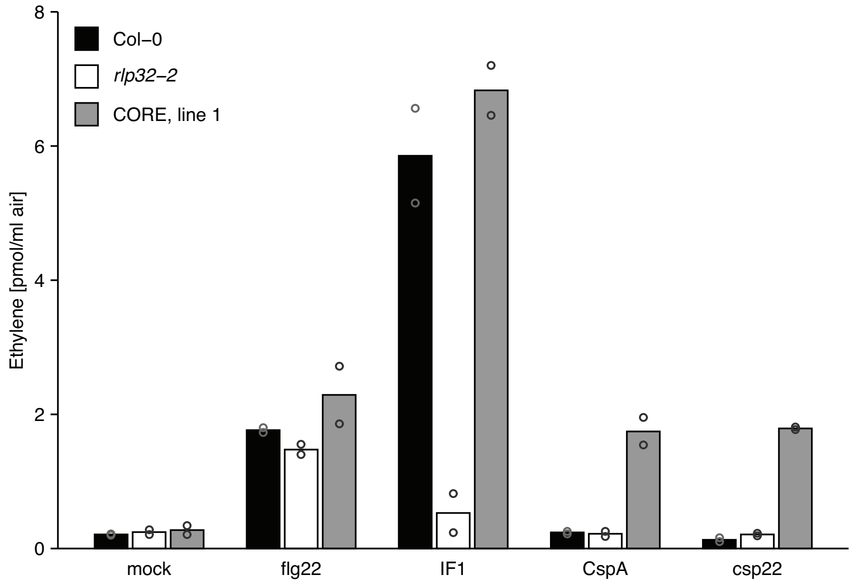

Supplementary Figure 16. **Bacterial cold shock protein CspA does not trigger ethylene production in *Arabidopsis*.** Col-0 wild-type plants, *rlp32* mutants or an *Arabidopsis* line stably expressing tomato cold shock receptor (CORE, line 1)[3] were treated with the elicitors indicated (100 nM) and assessed for ethylene production. Water (mock) treatment served as control. Data points indicate two replicates. Col-0 and *rlp32-2* plants were tested three times with similar results; two independent lines expressing CORE were tested and showed similar results.

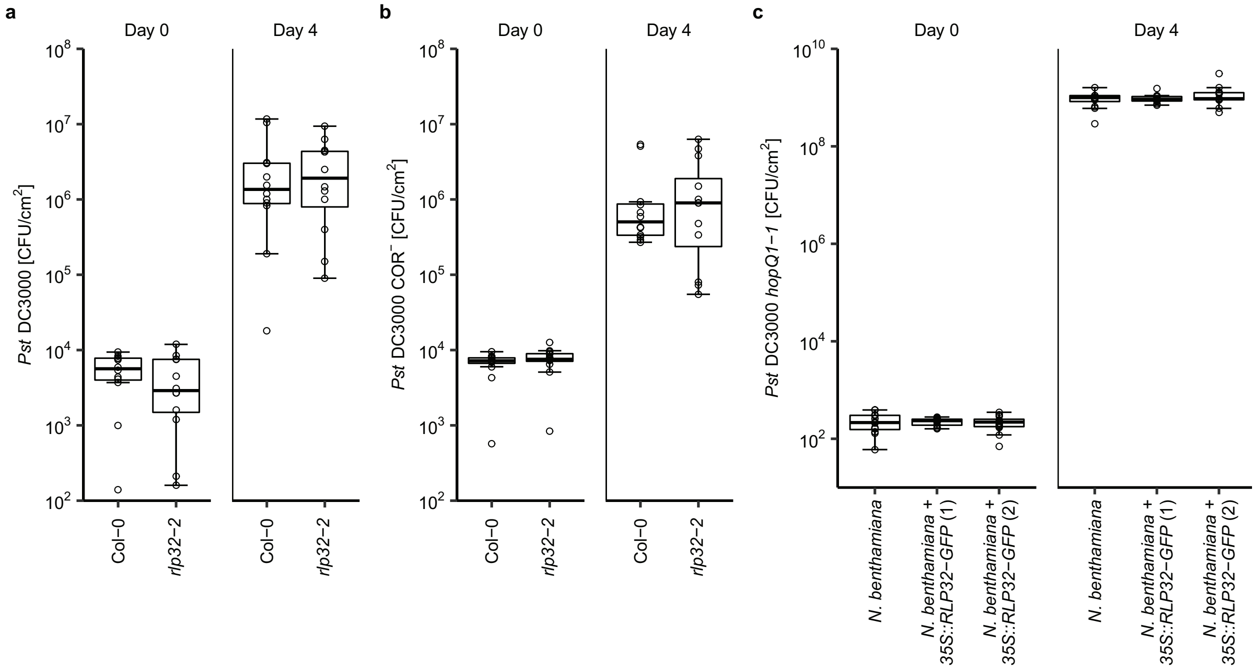

Supplementary Figure 17. **Impact of RLP32 on bacterial defense. a,b** Growth of *Pseudomonas syringae* pv. *tomato* DC3000 (*Pst*DC3000, spray inoculation, **a**) or the coronatine-deficient strain (*Pst*DC3000*COR*$^-$, spray inoculation, **b**) in *A. thaliana* Col-0 and *rlp32-2* plants. Bacterial growth was determined 0 and 4 days post inoculation. **c** Growth of *Pst*DC3000Δ*hopQ1-1* in wild-type or two *RLP32*-transgenic *N. benthamiana* lines at day 0 and 4 after infiltration (*n*=12 from 6 plants). For the box plots, centre line: median, bounds of box: 25th and the 75th percentiles, whiskers: 1.5 * IQR (IQR: the interquartile range between the 25th and the 75th percentile). No significant differences between Col-0 and *rlp32-2* plants (**a**, **b,** two-sided Mann-Whitney-U-test) or *N. benthamiana* wild-type and transgenic plants were observed (**c**, two-sided Steel test with *N. benthamiana* wild-type as control). Experiments were performed at least two times, with similar results.

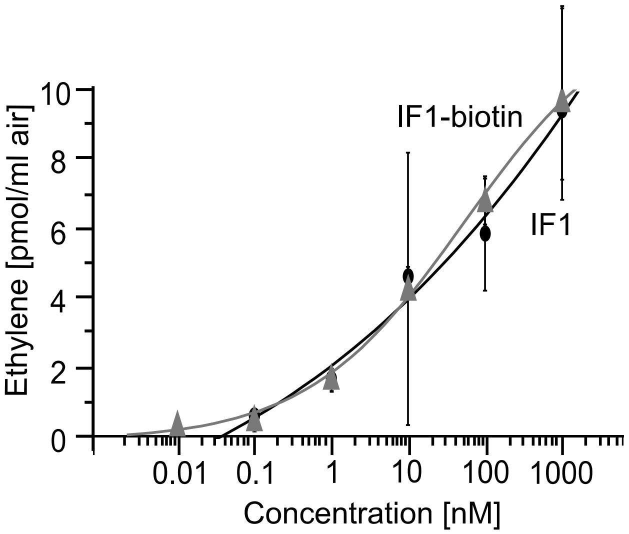

Supplementary Figure 18. **Biological activity of biotinylated IF1.** Ethylene accumulation in *Arabidopsis* Col-0 wild-type plants treated with IF1 recombinantly produced in *Pichia pastoris* (black) or treated with synthetic biotinylated IF1 (IF1-bio, grey). Data represent means ± SD of three replicates. The experiment was performed three times with similar results.

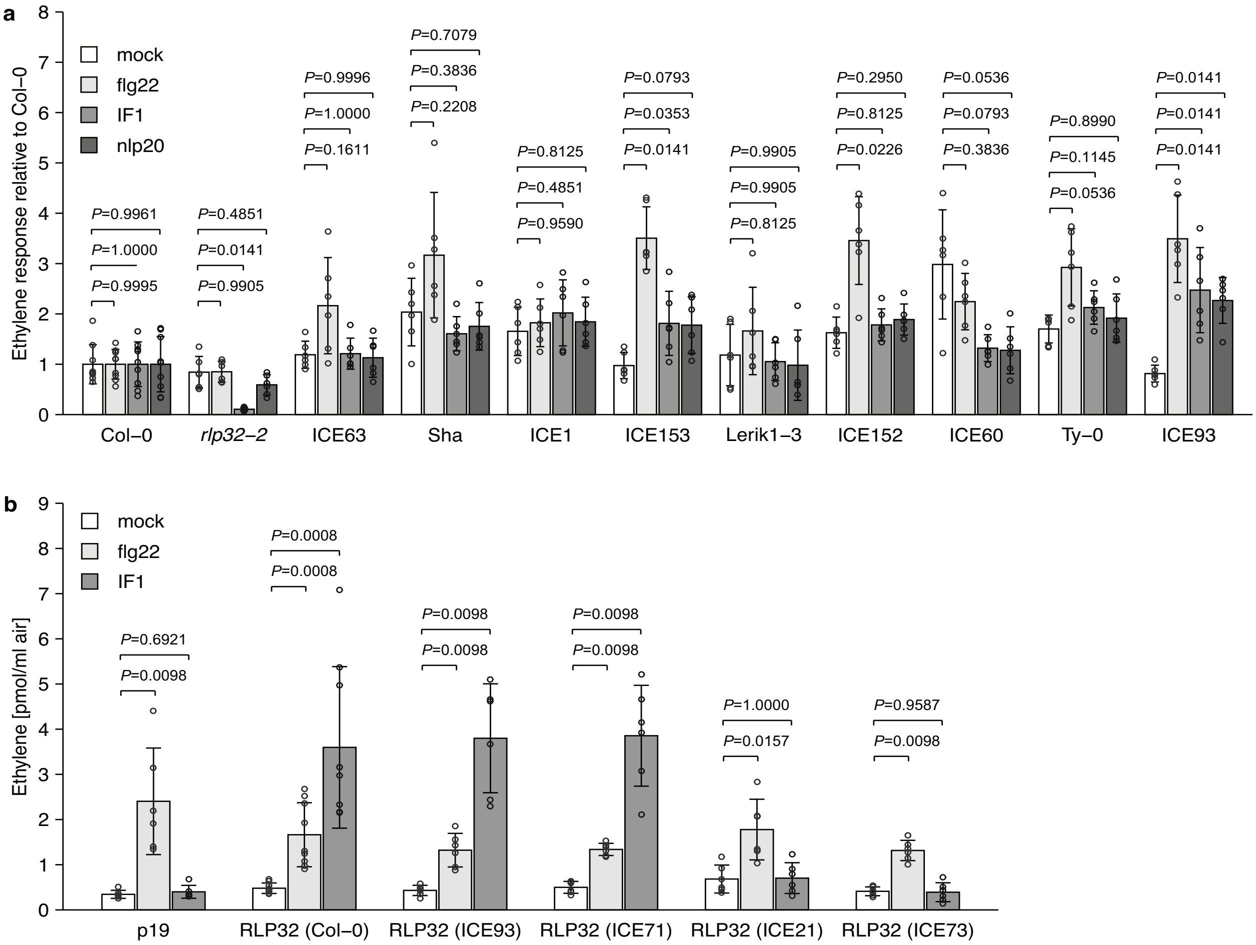

Supplementary Figure 19. **IF1 detection by RLP32 alleles from selected *Arabidopsis* accessions.** **a** Ethylene accumulation in *Arabidopsis* Col-0 wild-type plants, the *rlp32-2* mutant or indicated *Arabidopsis* accessions treated with water (mock), flg22, IF1 or nlp20. **b** Ethylene accumulation in *N. benthamiana* plants transiently expressing RLP32-GFP from indicated *Arabidopsis* accessions and treated with either water (mock), flg22 or IF1. Data represent means ± SD of 6 to 9 replicates. Statistically significant differences between mock and elicitor treatments were determined using a two-sided Steel test with mock treatment as control. Experiments were performed two times with similar results.

Supplementary Table 1. Primers used for genotyping.

line	Primer name	Primer sequence (5' – 3')
rlp32-2 (SM 3.3092)	L367	AATTGTTCAAAACCGGTTGTG
	R1406	CAGATTGAGTAGGGAAAGGGG
	Spm32	GAATAAGAGCGTCCATTTTAGAGTG
rlp32-3 (Salk_137467C)	L5	CGGAATTGAAGACGTTCGTT
	R994	TCACTGTTATTCGCCCATGA
	LBb1.3	ATTTTGCCGATTTCGGAAC
rlp32-4 (SM_3_33695)	L76	AAATTGGGCTGATAAAATGGG
	R1186	TCAATACAAGACGGGATTTGG
	Spm32	GAATAAGAGCGTCCATTTTAGAGTG
rlp32-5 (SM3_15851)	L528	TGTTGACAATTCAACGCAGAG
	R1697	AAATTTGGAAATGGATTTCGG
	Spm32	GAATAAGAGCGTCCATTTTAGAGTG

Supplementary Table 2. Primers and synthetic genes used for cloning.

Template	Expression in	Primer name	Primer sequence (5' – 3')
RLP32	*A. thaliana*, *N. benthamiana*	RLP32 endogenous promotor [-1597 bp] forward	GATTGCTTTGTGGAGTGGACTG
		RLP32-ATG forward	ATGAAAGACTCTTGGAACTCAACGAG
		RLP32 stop reverse	TTATTGCTTTCTCCTCAATCTTTTTTCATGTGC
		RLP32 no stop reverse	TTGCTTTCTCCTCAATCTTTTTTCATGTGC
IF1, *E. coli*		Start-forward	ATGGCCAAAGAAGACAATATTGAAATGCAAGG
		Stop reverse	TCAGCGACTACGGAAGACAATGCGG
		no stop reverse	GCGACTACGGAAGACAATGCGG
		I7 forward	ATGATTGAAATGCAAGGTACCGTTC
		I67 reverse	AATGCGGCCTTTGCTCAG
		+Cys N-terminal forward	ATGTGCGCCAAAGAAGACAATATTG
IF1, *E. coli*	*Pichia pastoris*	EcoRI_IF1_fwd	AATTGAATTCATGGCCAAAGAAGACAATATTGAAATGCAAGG
		NotI_IF1_nostop	AGAATTGCGGCCGCGCGACTACGGAAGACAATGCGG
		IF1_K39E_for	ACTGCACACATCTCCGGTGAAATGCGCAAAAACTACATCC
		IF1_K39E_rev	GATGTAGTTTTTGCGCATTTCACCGGAGATGTGTGCAGTAACC
		IF1_R41E_for	GCACACATCTCCGGTAAAATGGAAAAAAACTACATCCGCATCCT
		IF1_R41E_rev	AGGATGCGGATGTAGTTTTTTTCCATTTTACCGGAGATGTGTGC
		IF1_R41L_for	CACACATCTCCGGTAAAATGCTCAAAAACTACATCCGCATCC
		IF1_R41L_rev	AGGATGCGGATGTAGTTTTTGAGCATTTTACCGGAGATGTGTGC
		IF1_K39R41K42L_for	TTACTGCACACATCTCCGGTCTAATGCTCTTAAACTACATCCGCATCCTG
		IF1_K39R41K42L_rev	AGGATGCGGATGTAGTTTAAGAGCATTAGACCGGAGATGTGTGCAGT AACC
		IF1_R41pK42_for	ATCTCCGGTAAAATGCGCCCGAAAAACTACATCCGCATCC
		IF1_R41pK42_rev	ATGCGGATGTAGTTTTTCGGGCGCATTTTACCGGAGATG
IF1, *A. tumefaciens* C58		C58 forward	ATGTGCCGGGATTGTGTAG
		C58 no stop reverse	CTTGAAGCGATAGGTGATGC
IF1, *P. syringae*		DC3000 forward	ATGTCGAAAGAAGACAGCTTCGAAA
		DC3000 no stop reverse	ACGAGCGCGGTAGGTGAT
CspA	*Pichia pastoris*	Synthetic gene construct	gaattcATGTCCGGTAAAATGACTGGTATCGTAAAATGGTTCAACGCTGA CAAAGGCTTCGGCTTCATCACTCCTGACGATGGCTCTAAAGATGTGTTC GTACACTTCTCTGCTATCCAGAACGATGGTTACAAATCTCTGGACGAAG GTCAGAAAGTGTCCTTCACCATCGAAAGCGGCGCTAAAGGCCCGGCAG CTGGTAACGTAACCAGCCTGTAAgcggccgc
CspA-IF1-Helix	*Pichia pastoris*	Synthetic gene construct	gaattcATGTCAGGGAAAATGACAGGAATCGTTAAGTGGTTCAATGCTG ACAAAGGCTTTGGCTTCATTACTCCAGATGATGGTAGTAAGGACGTAT TTGTGCATTTCTCTGCCATTCAATCCGGAAAGATGAGAAAGAACTACC TTGATGAAGGTCAGAAAGTCTCGTTTACCATAGAGTCTGGAGCTAAA GGTCCTGCAGCTGGTAACGTTACTAGCTTGgcggccgc
IF1, *A. thaliana*	*Pichia pastoris*	Synthetic gene construct	gaattcATGATTAGGTGTCAGAGGGCATCAGGAGGTCGGGGCGGGGCG AATAGAAGCAAACCGGCTAAACCTCAAGTCAAGGAAGGCAGTAACAAG ACAGTGATTGAGGGATTAGTTACGGAAAGTCTTCCAAACGGTATGTTTC GAGTAGATCTCGAAAACGGTGACA ATATATTGGGGTACATTTGCGGAA AAATCCGGAAGAATTTTATCCGAATCCTGCCCGGGGATAAGGTAAAAG TTGAGATGTCTGTGTATGACTCGACTAAAGGCCGCATAATATTCCGTAT GTCATCCAGAGATgcggccgc

Supplementary Table 3. Primers used for qRT-PCR

Primer name	Primer sequence (5' – 3')
qEF1a_F	GAGGCAGACTGTTGCAGTCG
qEF1a_R	TCACTTCGCACCCTTCTTGA
qFRK1_F	AAGAGTTTCGAGCAGAGGTTGAC
qFRK1_R	CCAACAAGAGAAGTCAGGTTCGTG
qPAD3_F	CGAGCATCTTAAGCCTGGAA
qPAD3_R	ACTCCACCAATCCCTGCTAC
qPDF1.2_F	GCCAAGTGGGACATGGTCAG
qPDF1.2_R	GGGACGTAACAGATACACTTG

References

1 Wang, G. *et al.* A genome-wide functional investigation into the roles of receptor-like proteins in Arabidopsis. *Plant Physiol* **147**, 503-517 (2008).

2 Sette, M. *et al.* The structure of the translational initiation factor IF1 from E-coli contains an oligomer-binding motif. *Embo J* **16**, 1436-1443 (1997).

3 Wang, L. *et al.* The pattern-recognition receptor CORE of Solanaceae detects bacterial cold-shock protein. *Nat Plants* **2**, 16185 (2016).
