# Supplementary material for: Genotyping-by-sequencing-based identification of Arabidopsis pattern recognition receptor RLP32 recognizing proteobacterial translation initiation factor IF1
Source: Nat Commun. 2022 Mar 11;13:1294. doi: 10.1038/s41467-022-28887-4 (PMC8917236; doi:10.1038/s41467-022-28887-4)
Supplement: Supplementary file 3 — Reporting Summary [file 41467_2022_28887_MOESM3_ESM.pdf]

nature portfolio

Corresponding author(s): DBPR NCOMMS-21-11917

Last updated by author(s): Jan 28, 2022

Reporting Summary

Nature Portfolio wishes to improve the reproducibility of the work that we publish. This form provides structure for consistency and transparency in reporting. For further information on Nature Portfolio policies, see our Editorial Policies and the Editorial Policy Checklist.

Statistics

For all statistical analyses, confirm that the following items are present in the figure legend, table legend, main text, or Methods section.

n/a	Confirmed	
☐	☒	The exact sample size (*n*) for each experimental group/condition, given as a discrete number and unit of measurement
☐	☒	A statement on whether measurements were taken from distinct samples or whether the same sample was measured repeatedly
☐	☒	The statistical test(s) used AND whether they are one- or two-sided<br>*Only common tests should be described solely by name; describe more complex techniques in the Methods section.*
☒	☐	A description of all covariates tested
☒	☐	A description of any assumptions or corrections, such as tests of normality and adjustment for multiple comparisons
☐	☒	A full description of the statistical parameters including central tendency (e.g. means) or other basic estimates (e.g. regression coefficient) AND variation (e.g. standard deviation) or associated estimates of uncertainty (e.g. confidence intervals)
☐	☒	For null hypothesis testing, the test statistic (e.g. F, t, r) with confidence intervals, effect sizes, degrees of freedom and P value noted<br>*Give P values as exact values whenever suitable.*
☒	☐	For Bayesian analysis, information on the choice of priors and Markov chain Monte Carlo settings
☒	☐	For hierarchical and complex designs, identification of the appropriate level for tests and full reporting of outcomes
☒	☐	Estimates of effect sizes (e.g. Cohen's *d*, Pearson's *r*), indicating how they were calculated

Our web collection on statistics for biologists contains articles on many of the points above.

Software and code

Policy information about availability of computer code

Data collection	*Provide a description of all commercial, open source and custom code used to collect the data in this study, specifying the version used OR state that no software was used.*
Data analysis	RAD-seq/QTL mapping package R/qtl (https://rqtl.org)<br>Protein sequence analysis (http://signal.salk.edu/atg1001/3.0/gebrowser.php)<br>Statistical data analysis JMP (15.2.0 and 16.0.0; SAS Institute Inc., Cary, NC, US) and MS Office Excel (4.1.0)<br>iterative threading assembly refinement (I-TASSER)-based 3-dimensional structure prediction (https://zhanggroup.org/I-TASSER/)<br>determination of protein concentration protparam tool (http://web.expasy.org/protparam)<br>Protein domain prediction UniProt database (https://www.uniprot.org)<br>Phylogenetic tree construction MEGA X (https://www.megasoftware.net/)<br>quantification of callose pixels ImageJ (https://imagej.nih.gov/ij/)<br>protein sequence alignment and conservation score determination Easy Sequencing in PostScript (ESPript 2.2, http://espript.ibcp.fr/ESPript/ESPript/), PYMOL (https://pymol.org/2/)<br>MaxQuant software, version 1.5.2.8 (https://www.maxquant.org)<br>SHORE software (https://1001genomes.org/software/shore.html)<br>Burrows-WheelerAligner software BWA (http://bio-bwa.sourceforge.net)

For manuscripts utilizing custom algorithms or software that are central to the research but not yet described in published literature, software must be made available to editors and reviewers. We strongly encourage code deposition in a community repository (e.g. GitHub). See the Nature Portfolio guidelines for submitting code & software for further information.

Data

Policy information about availability of data

All manuscripts must include a data availability statement. This statement should provide the following information, where applicable:

- Accession codes, unique identifiers, or web links for publicly available datasets
- A description of any restrictions on data availability
- For clinical datasets or third party data, please ensure that the statement adheres to our policy

All data are available within this article and its Supplementary Information. RLP32 amino acid sequences from A. thaliana accessions were obtained from the 1001 Genomes project (http://signal.salk.edu/atg1001/3.0/gebrowser.php). Source data are provided with this paper. Original gel blots are shown in the Source data file. RAD-seq data have been deposited to the Dryad repository with the dataset identifier doi:10.5061/dryad.h70rxwdkx. This information, which will be made publicly available upon acceptance of this MS, can be temporarily accessed via the following hyperlink: https://datadryad.org/stash/share/2GYkEuBAhzJ869oP8vt4NDhd8eKfpmbHZt9OOVQ9XU8.
Mass spectrometry proteomics data have been deposited to the ProteomeXchange Consortium via the PRIDE59 partner repository with the data set identifier PXD031124 using the login information below. This information will be made publicly available upon acceptance of the MS.
Username: reviewer_pxd031124@ebi.ac.uk
Password: k6avHgHR

Field-specific reporting

Please select the one below that is the best fit for your research. If you are not sure, read the appropriate sections before making your selection.

☒ Life sciences ☐ Behavioural & social sciences ☐ Ecological, evolutionary & environmental sciences

For a reference copy of the document with all sections, see nature.com/documents/nr-reporting-summary-flat.pdf

Life sciences study design

All studies must disclose on these points even when the disclosure is negative.

Sample size	Sample size was determined based on experimental trials and previously published studies. No statistical methods were used to predetermine sample size. Previous publications considered to determine sample size include:<br><br>Ethylene production (Albert, I., et al. An RLP23–SOBIR1–BAK1 complex mediates NLP-triggered immunity. Nature Plants. 1, 15140 (2015))<br>ROS burst (Wan, W. L. et al. Comparing Arabidopsis receptor kinase and receptor protein-mediated immune signaling reveals BIK1-dependent differences. New Phytol. 221, 2080-2095, (2019))<br>Callose quantification (Kim M.G., Mackey D. Measuring Cell-Wall-Based Defenses and Their Effect on Bacterial Growth in Arabidopsis. Innate Immunity, 443-452, (2008))<br>Gene expression analysis (Wan, W. L. et al. Comparing Arabidopsis receptor kinase and receptor protein-mediated immune signaling reveals BIK1-dependent differences. New Phytol. 221, 2080-2095, (2019))<br>Resistance pathoassays (Böhm, H. et al. A conserved peptide pattern from a widespread microbial virulence factor triggers pattern-induced immunity in Arabidopsis. PLoS Pathog. 10, e1004491, (2014))
Data exclusions	No data were exluded from the analyses provided.
Replication	Reproducibility of data was tested by multiple repetitions of the experiments described. All experiments were conducted at least 3 times on different days using biological materials produced independently (biological replicates) if not stated otherwise. At least 3 technical replicates were included in the individual biological replicate experiments if not stated otherwise. Statistical evaluation was applied to all data sets obtained and is mentioned in figure legends when applicable. All attempts at replication were successful.
Randomization	Allocation of test plants used in our study was random. There was no targeted selection of individual plants for specific treatments.
Blinding	Blinding was not used in our study as it does not include clinical trials. The nature of the experiments conducted in our study requires that the experimentator knows precisely what plants have received what treatment. In plant, biology blinded/double-blinded studies are uncommon.

Reporting for specific materials, systems and methods

We require information from authors about some types of materials, experimental systems and methods used in many studies. Here, indicate whether each material, system or method listed is relevant to your study. If you are not sure if a list item applies to your research, read the appropriate section before selecting a response.

Materials & experimental systems

n/a	Involved in the study
☐	☒ Antibodies
☒	☐ Eukaryotic cell lines
☒	☐ Palaeontology and archaeology
☒	☐ Animals and other organisms
☒	☐ Human research participants
☒	☐ Clinical data
☒	☐ Dual use research of concern

Methods

n/a	Involved in the study
☒	☐ ChIP-seq
☒	☐ Flow cytometry
☒	☐ MRI-based neuroimaging

Antibodies

Antibodies used

Streptavidin-alkaline phosphatase conjugate, Roche, Mannheim, Germany, Cat.-No. 11089161001, Lot 42821400
anti-phospho-p44/42-MAPK (Erk1/2), Cell Signalling Technology Europe, Frankfurt, Germany, Cat.-No. 9101, Lot-No. 31
anti-GFP, Torrey Pines Biolabs, Secaucus, New Jersey, US, Cat.-No. TP401, Lot 081211
anti-HA, Sigma-Aldrich, St. Louis, Missouri, US, Cat.-No. H3663, Lot 066M4837V3
anti-Myc, Sigma-Aldrich, St. Louis, Missouri, US, Cat.-No. C3956, Lot 094M4775V

Secondary antibodies
anti-Mouse IgG–AP antibody produced in rabbit, Sigma-Aldrich, St. Louis, Missouri, US, Cat.-No. A4312, Lot 091M4753
anti-Rabbit IgG–AP antibody produced in goat, Sigma-Aldrich, St. Louis, Missouri, US, Cat.-No. A3687, Lot 067K6071

Dilution used
Streptavidin 1:2500
phospho-p44/42-MAPK 1:3000
anti-GFP: 1:4000
anti-HA: 1:2000
anti-Myc: 1:5000
anti-Mouse: 1:10000
anti-Rabbit: 1:10000

Validation

Validation information and experiments can be obtained from the following websites:
Streptavidine-alkaline phosphatase conjugate https://www.sigmaaldrich.com/DE/de/product/roche/11089161001?gclid=EAIaIQobChMI8_a2qLXS9QIVi-R3Ch3nHQ-iEAAYAiAAEgJlzPD_BwE
p42/44 https://www.cellsignal.de//products/primary-antibodies/p44-42-mapk-erk1-2-antibody/9101
Myc https://www.sigmaaldrich.com/content/dam/sigma-aldrich/docs/Sigma/Datasheet/3/c3956dat.pdf
GFP (Torrey Pines) http://www.chemokine.com/Houston/rat&other/GFP.PDF
HA (Sigma) https://www.sigmaaldrich.com/content/dam/sigma-aldrich/docs/Sigma/Datasheet/2/h3663dat.pdf
anti-Mouse IgG-AP antibody produced in rabbit https://www.sigmaaldrich.com/DE/en/product/sigma/m7023
anti-Rabbit IgG-AP antibody produced in goat https://www.sigmaaldrich.com/DE/en/product/sigma/r3128?gclid=EAIaIQobChMI3_2EwrbS9QIVB7d3Ch04ig9LEAAYASAAEgKqOPD_BwE
